# Clinical and genetic studies for a cohort of patients with Leber congenital amaurosis

**DOI:** 10.1007/s00417-024-06450-9

**Published:** 2024-04-25

**Authors:** Yunyu Zhou, Lijuan Huang, Yan Xie, Wen Liu, Shasha Zhang, Lili Liu, Ping Lin, Ningdong Li

**Affiliations:** 1 Department of Ophthalmology, Beijing Children’s Hospital, Capital Medical University, Beijing, 100045 China; 2 Department of Ophthalmology, The Second Affiliated Hospital of Fujian Medical University, Quanzhou, 362000 China; 3 Department of Ophthalmology, Xi’an Children’s Hospital, Xi’an, 710002 China; 4 Department of Ophthalmology, Shanghai General Hospital, Shanghai Jiao Tong University School of Medicine, Shanghai, 200940 China

**Keywords:** LCA, Gene, Retinal vasculature

## Abstract

**Purpose:**

Leber congenital amaurosis (LCA) is a group of early-onset retinal degenerative disorders, resulting in blindness in children. This study aimed to describe the clinical and genetic characteristics of a cohort of patients with LCA and to investigate the retinal vascular characteristics in LCA patients.

**Methods:**

Fifty-two children with LCA were included in the study. All patients underwent detailed ocular examinations. Electroretinography (ERG) was used to evaluate the retinal function. Optical coherence tomography (OCT) was used to assess the structure change of the retina for those patients who were able to cooperate very well. Panel-based next-generation sequencing was performed to identify pathogenic variants in genes associated with LCA. Diameters of the retinal vessels were measured using the EVision AI screening system with an artificial intelligence (AI) technique. An ultrasound Doppler was used to evaluate hemodynamic parameters, including peak systolic velocity (PSV), resistive index (RI), and pulsatility index (PI), in the ophthalmic, central retinal, posterior ciliary, carotid, and internal carotid as well as external carotid arteries in 12 patients aged from 3 to 14 years.

**Results:**

We detected 75 pathogenic variants from ten genes of *RPGRIP1*, *CEP290*, *GUCY2D*, *LCA5*, *AIPL1*, *CRB1*, *RPE65*, *CRX*, *RDH12*, and *TULP1*, including 29 novel and 36 previously reported variants in 52 affected children with LCA, with the highest detective rate in *RPGRIP1* (26.9%). Fundus appearance is diverse in patients with LCA, ranging from normal to severe peripheral or central retinopathy. Retinal vasculature was evaluated in 12 patients with different gene variants, showing narrowed arteries with an average diameter of 43.6 ± 3.8 μm compared to that of 51.7 ± 2.6 μm in the normal controls (*P* < 0.001, *n* = 12). Meanwhile, their hemodynamic parameters were changed as well in the ophthalmic artery (OA), with a decreased PSV (*P* = 0.0132, *n* = 12) and slightly increased PI (*P* = 0.0488, *n* = 12) compared to the normal controls. However, the hemodynamic parameters did not change significantly in the other vessels.

**Conclusions:**

Blood supply to the eyeball is predicted to be reduced in patients with LCA, presumably due to photoreceptor cell degeneration. The novel identified variants will expand the spectrum of variants in LCA-related genes and be useful for studying the molecular mechanisms of LCA.

**Supplementary Information:**

The online version contains supplementary material available at 10.1007/s00417-024-06450-9.


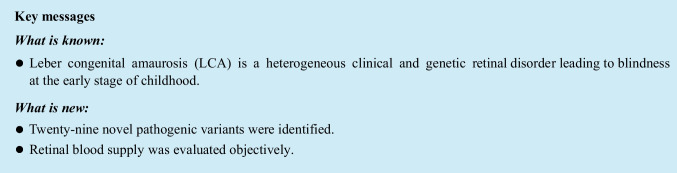


## Introduction

Leber congenital amaurosis (LCA, MIM204000) is a group of inherited retinal disorders (IRD) with an estimated prevalence of 1 in 33,000–81,000 in the general population [1]. It is the leading cause of early-onset visual impairment, typically in the first few months of life. People with LCA may have multiple clinical manifestations, including low vision, poor fixation and pursuit, nystagmus, photophobia, nyctalopia, sluggish pupillary response to light, and strabismus [1]. Fundus may present with a variety of characteristics, ranging from a normal appearance to severe retinopathy, which manifests as pallor or edema in the optic disc, narrowing of the retinal blood vessels, pigment deposition in the retina, and/or maculopathy. OCT examination has revealed that retinal layers usually atrophy with the rupture of the ellipsoidal layer. Electroretinography (ERG) recording shows reduced a- and b-waves or extinguished a- and b-waves [2].

LCA can be inherited in either an autosomal recessive (AR) or an autosomal dominant (AD) pattern, with the former being more common and the latter being rare [2]. Currently, there have been 25 disease-causing genes identified in association with LCA, with 23 of them being inherited in an AR manner, and only three genes (*CRX*, *IMPDH1*, and *OTX2)* being inherited in an AD manner (https://sph.uth.edu/retnet/sum-dis.htm). These genes are expressed primarily in the retina and are involved in maintaining the normal structure and function of a healthy retina. For example, the genes of *GUCY2D*, *AIPL1*, *RD3*, and *KCNJ13* are involved in the phototransduction cascade, and the genes of *RPE65*, *LRAT*, and *RDH12* play important roles in retinoid cyclic metabolism. Transportation in the cilia relies on proteins encoded by the genes of *LCA5*, *RPGRIP1*, *CEP290*, *TULP1*, *SPATA7*, and *IQCB1*. Meanwhile, photoreceptor morphogenesis is determined by the normal function of the *CRX*, *CRB1*, *GDF6*, and *PRPH2* genes, and photoreceptor differentiation is related to the *OTX2*. In addition, *IMPDH1* is involved in guanine synthesis in the retina. Mutations in these genes will lead to the degeneration of the photoreceptor cell [3, 4].

Due to the clinical and genetic heterogeneity of LCA, a correct diagnosis of LCA is often established based on detailed medical history, retinal photography and OCT images, ERG examination, and genetic testing. However, to the best of our knowledge, several clinical phenomena of LCA have yet to be plausibly explained, for example, the causal relationship between photoreceptor degeneration and vessel attenuation, and the condition of the blood supply to the eyeball and especially to the retina and choroid. We previously attempted to assess the blood supply to the retina and choroid using OCT angiography (OCTA) but failed due to nystagmus and poor fixation. Recently, color Doppler imaging (CDI), as a rapid and noninvasive technology, has been used to evaluate the hemodynamics in numerous retinal disorders including age-related macular degeneration (AMD) and retinitis pigmentosa (RP) ​​[5–7]. In this study, we aimed to characterize the clinical characteristics of 52 Han Chinese patients with LCA and evaluate the vascular system in the retina and blood supply to the eyes of 12 patients with different mutations using CDI and AI techniques.

## Methods

### Patients

A total of 52 patients from 49 unrelated families were enrolled in the study. All underwent careful ocular examination, including the anterior segment, fundus, and refractive status. Fundus photography was taken using a Canon camera (Kowa, Tokyo, Japan) and/or Optos imaging system (Optos, Gaush Meditech Ltd, China). The RetCam system (Clarity Medical Systems Inc., Pleasanton, CA) was used as well for those uncooperative children. Retinal structure was evaluated for cooperative children using the spectral domain optical coherence tomography (SD-OCT, Heidelberg Engineering, Heidelberg, Germany). The retinal pigment epithelial (RPE) layer was assessed using autofluorescence imaging (FAF, Heidelberg Engineering, Heidelberg, Germany). Full-field ERGs were recorded using the RETI-scan 21 system (Roland company, Germany) following the guidelines set by the International Society for Clinical Electrophysiology of Vision (http://www.iscev.org). Cycloplegic refraction was performed on cooperative children over the age of 2 years. Autorefraction was performed using a handheld autorefractor (Welch Allyn VS100, China) for the uncooperative children. The refractive status was denoted using the spherical equivalent (SE) measured in diopters (D). Multidisciplinary consultations were conducted to access disorders in different systems, including sensory hearing loss, diabetes, neurological, cardiac, and renal disorders. Moreover, genetic testing was performed to aid molecular diagnosis. This study adhered to the tenets of the Declaration of Helsinki and was approved by the Ethics Committee of the Beijing Children’s Hospital (2022-E-213-R).

### Genetic screening and bioinformatics analysis

Genomic DNA was extracted following a standardized protocol provided by Roche Biochemical, Inc. Next-generation sequencing was commercially performed by the Mygenomic Biochemical Company (Beijing, China). The DNA library was constructed and sequenced on an Illumina HiSeq X Ten platform (Illumina, San Diego, CA) for the paired reads of 150 bp. The clean reads (< 150 bp) were aligned to the UCSC hg19 human reference genome after eliminating duplicated and low-quality reads. Variants were analyzed using the Genome Analysis Toolkit (GATK) program, with a GATK-assigned quality criterion score of more than 50.0 to be considered for further analyses. Variants were annotated by the ANNOVAR software (http://annovar.openbioinformatics.org/en/latest/), combined with the public databases including 1000 genome, ESP6500, dbSNP, genomAD, ClinVar, and one in-house database of MyGenostics. Segregation analysis was performed in all cases. Clone sequencing was performed for the indel variant. Large deletions were verified by real-time quantitative PCR (qPCR). Primer sequences used for clone sequencing and qPCR are listed in Supplementary Table 1 and 2. Pathogenicity analysis of missense variant was performed using online tools including polyPhen-2 (http://genetics.bwh.harvard.edu/pph2/), Sorting Intolerant from Tolerant (https://sift.bii.a-star.edu.sg/, Protein Analysis), and PROVEAN (http://provean.jcvi.org/index.php). The splicing site variant was analyzed by the SpliceAI program (https://github.com/Illumina/SpliceAI). Pathogenicity was predicted based on the guidelines of the American College of Medical Genetics and Genomics (ACMG). Mutations were named following the nomenclature recommended by the Human Genomic Variation Society (HGVS). Multiple sequence alignments were performed using the GENEDOC software. Sequence logos were generated using WebLogo3 (http://weblogo.threeplusone.com/). The three-dimensional structure of the protein was modelled in the AlphaFold Protein Structure Database (https://alphafold.ebi.ac.uk/entry/Q02846), and visualized using the PyMol program (https://pymol.org/2/). The stability of protein was predicted by the DynaMut program (https://biosig.lab.uq.edu.au/dynamut/).

### Measurement of blood vessels and blood flow

The diameter of the retinal vessels was evaluated using the EVision AI screening system (EVision AI, Beijing, China, https://yiweiimage.com) on the standard 45° fundus photos. After delineating a region of interest (ROI), denoising, normalizing, and sharpening the vascular boundaries, the pre-processed fundus images were inputted into the ResUNet deep learning network [8]. The distinction between artery and vein vessels is determined by their respective brightnesses and colors. The diameter of the vessel is automatically calculated after segmentation and extraction in the EVision AI system (Supplement Fig. 1).

**Fig. 1:**
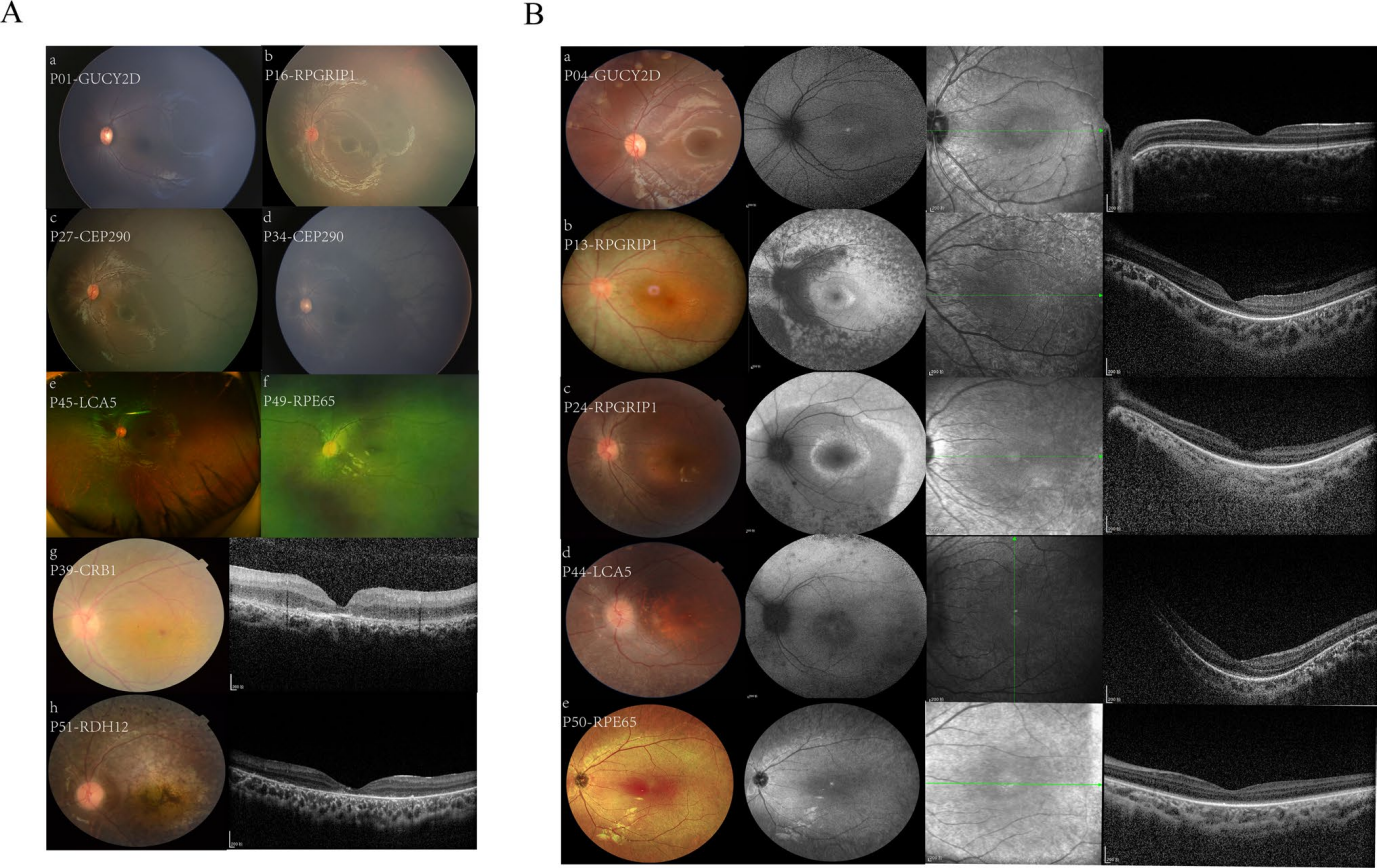
Different manifestations of fundus and retinal structure in different causative genes of LCA. **A**(a) **B**(a) Patient 01 (5 months) and Patient 04 (6 years old) with the mutations of *GUCY2D* showed an almost normal fundus appearance. **A**(b) Patient 16 (2-year-old) with the mutations of *RPGRIP1* showed attenuated vessels and mottled fundus. **A**(c, d) Patient 27 (1-year-old), 34 (5 months) with the mutations of *CEP290* showed patchy retinal degeneration in the peripheral retina. **A**(e) Patient 45 with the mutations of *LCA5* (3-year-old) showed thinner retina and periphery retinal pigment rearrangement. **A**(f) Patient 49 (5-year-old) with the mutations of *RPE65* showed a white dot deposit. **A**(g) Patient 39 (3-year-old) with the mutations of *CRB1* had the gold-foil reflection of macular, and the OCT image showed a coarsely laminated retina. **A**(h) Patient 51 (5-year-old) with the mutations of *RDH12* showed attenuated vessels, macular atrophy, and bone-spicule pigmentation. OCT revealed marked atrophy of the outer retinal layer in the macular region, with the evident decline of the outer nuclear layer thickness and loss of the external limiting membrane and ellipsoid zone. **B**(b, c) Patient 13 (14 years old) with the mutations of *RPGRIP1* showed a grey optic disc, attenuated vessels, retinal exudated, and retinal pigment deposited. Patients 24 (6 years old) showed retinal pigment rearrangement. **B**(d) Patient 44 (6 years old) with the mutations of *LCA5* showed pale optic nerve and pigmentary changes. **B**(e) Patient 50 (5 years old) with the mutations of *RPE65* showed mottled fundus. RPE atrophy, OCT scans confirmed that the peripheral outer retinal layers (myoid, ellipsoid, and outer segment layer) were thinned and disappeared in Figure **B**(b, c, d, e)

Hemodynamic parameters of the ocular artery (OA), central retinal artery (CRA), posterior ciliary artery (PCA), carotid artery (CCA), internal carotid artery (ICA), and external carotid artery (ECA) were measured using Doppler ultrasound (MyLab™Twice ultrasound system, Esaote Genoa, Italy), including peak systolic velocity (PSV), end-diastolic velocity (EDV), resistance index (RI), and pulse index (PI). The formula for RI is RI = PSV − EDV/PSV and for PI is PI = PSV − EDV/mV, where mV is the mean blood flow velocity.

### Statistical analysis

An independent *t*-test was used to compare the difference in the vascular and blood flow parameters between the LCA patients and the normal controls, using the statistical software package of SPSS version 23 (SPSS, version 23.0; IBM, Armonk, NY, USA), with a *P*-value of less than 0.05 to be considered statistically significant.

## Results

### Demographics and clinical characteristics

​Fifty-two affected children, 32 boys and 20 girls, were evaluated in the study. The age of the patients ranged from 4 months to 14 years, with a median age of 4.1 ± 3.1 years at the initial visit to our clinic. All patients presented with low vision, poor fixation, and nystagmus. Additional clinical findings were ocular-digital signs in 20 patients, photophobia in 14 patients, strabismus in nine patients, night blindness in four patients, and cataracts in two patients. The affected children who were able to cooperate with vision tests demonstrated reduced visual acuity, ranging from light perception to 20/100. Most of the patients were hyperopia, except two people who had myopia.

Except for the common feature of attenuated blood vessels in the retina, the appearance of the fundus in our patients ranged from a near-normal appearance to severe retinopathy. For example, our patients with the *GUCY2D* variant had a roughly normal appearance in their fundi, so one patient was misdiagnosed as “hyperopic, amblyopia, and nystagmus” for a long time until 14 years old. Patients with the *RPGRIP1* variant exhibited a mottled appearance in their fundi. OCT scans revealed that their retinas were thinned and that the outer layers, including the myoid, ellipsoid, and outer segmental layers, had disappeared at the periphery of the retina. Patients with the *CEP290* variant had a patchy appearance on the periphery of the retina. Patients with the *CRB1* variant revealed a gold-foil reflection in the macular region. The OCT scan revealed a coarsely laminated retina. Patients with the *LCA5* variant had a flecked appearance to their fundi. OCT scans showed that the RPE layer was atrophied and disrupted, which was supported by autofluorescence images showing fluorescence losses. Patients with variant *RPE65* had a mottled fundus appearance, with one patient showing deposits of white spots in his fundus. OCT scans revealed that their retinas were thinned. One patient with the *RDH12* variant showed macular atrophy and bone-like pigment deposits in his fundus. The OCT scan revealed that the retinal structure was disrupted and the entire retina was thinned (Fig. 1A, 1B, and Table 1). ERG recordings were extinguished in all patients under both photopic and scotopic conditions. The detailed clinical features are listed in Supplement Table 3.

**Table 1:** Phenotype-genotype associations among Leber congenital amaurosis patients

Gene	Number of patient(s) (male/female)	Mean of age (years)	Mean age of onset (months)	VA in the better-seeing eye	Refraction	Cataract	Strabismus	Fundus
*GUCY2D*	8 (3/5)	3.4 ± 4.7	3.3 ± 1.2	20/400	Hyperopia	25% (2/8)	12.5(1/8)	AV
*AIPL1*	4 (1/3)	2.8 ± 1.7	5.5 ± 2.1	CF	Hyperopia	0	0	RPR
*RPGRIP1*	14 (8/6)	4.7 ± 3.6	4.1 ± 2.4	20/125	Hyperopia, myopia	0	14.3% (2/14)	BMD
*CEP290*	10 (9/1)	2.8 ± 1.7	3.2 ± 1.8	20/200	Hyperopia	0	20% (2/10)	PRD
*CRB1*	4 (3/1)	4 ± 1.4	10 ± 2.8	20/100	Hyperopia	0	25% (1/4)	MD
*CRX*	1 (M = 1)	1	1	LP	NA	0	0	NA
*LCA5*	5 (3/2)	6 ± 3.1	5 ± 0.7	20/100	Hyperopia	0	40% (2/5)	RPR
*RPE65*	4 (3/1)	5 ± 1.6	4 ± 0.8	20/100	Hyperopia	0	0	BMD, WDD
*RDH12*	1 (F = 1)	5	6	20/250	Hyperopia	0	0	BSP, MD
*TULP1*	1 (M = 1)	6	3	20/200	NA	0	100% (1/1)	NA

*VA*, visual acuity; *CF*, count fingers; *LP*, light perception; *AV*, attenuated vessels; *RPR*, retinal pigment rearrangement; *BMD*, bilateral mottled fundus; *PRD*, patchy retinal degeneration; *WDD*, white-dot deposition; *BSP*, bone-spicule pigmentation; *MD*, macular degeneration

### Pathogenic variants

Seventy-five variants were detected in our patients, including 29 novel and 46 previously reported variants in nine LCA-associated genes of *RPGRIP1*, *CEP290*, *GUCY2D*, *LCA5*, *AIPL1*, *RPE65*, *CRB1*, *CRX*, *RDH12*, and *TULP1*, with the highest detection rate of 26.9% (14/52) in *RPGRIP1*, followed by *CEP290* (19.2%, 10/52), *GUCY2D* (15.4%, 8/52), *LCA5* (9.6%, 5/52), *AIPL1* (7.7%, 4/52), *RPE65* (7.7%, 4/52), *CRB1* (7.7%, 4/52), *CRX* (1.9%, 1/52), *RDH12* (1.9%, 1/52), and TULP1 (1.9%, 1/52) (Supplement Fig. 2A). Thirty-eight compound heterozygous variants and 11 homozygotes variants were detected in our patients, in which 13 compound heterozygous variants were found in *RPGRIP1*, 10 in *CEP290*, six in *GUCY2D*, three each in *AIPL1* and *CRB1*, two each in *LCA5* and *RPE65*, and one in *TULP1*, and three homozygous variants were detected in *LCA5*, two of them in *GUCY2D* and *RPE65*, and one in *RPGRIP1*, *AIPL1*, *CRB1*, and *RDH12*, separately. Only one patient carried a heterozygous variant of c.571delT (p.Y191Mfs*3) in the *CRX* gene. All above variants included 24 missense, 15 nonsense, 27 indels, and nine splicing site variants (Supplement Fig. 2B). The variants of c.535delG (p.E179Sfs*11) in *RPGRIP1* and c.421C > T (p.Q141X) in *AIPL1* presented recurrently in six times and four times respectively in non-consanguineous patients. All variants and their calculated scores using the programs of SIFT, Polyphen-2, Provean, and Splice AI, as well as the ACMG classification, are listed in Supplement Table 4. Pedigrees of the families with novel variants in LCA genes are shown in Supplement Fig. 3.

**Fig. 2:**
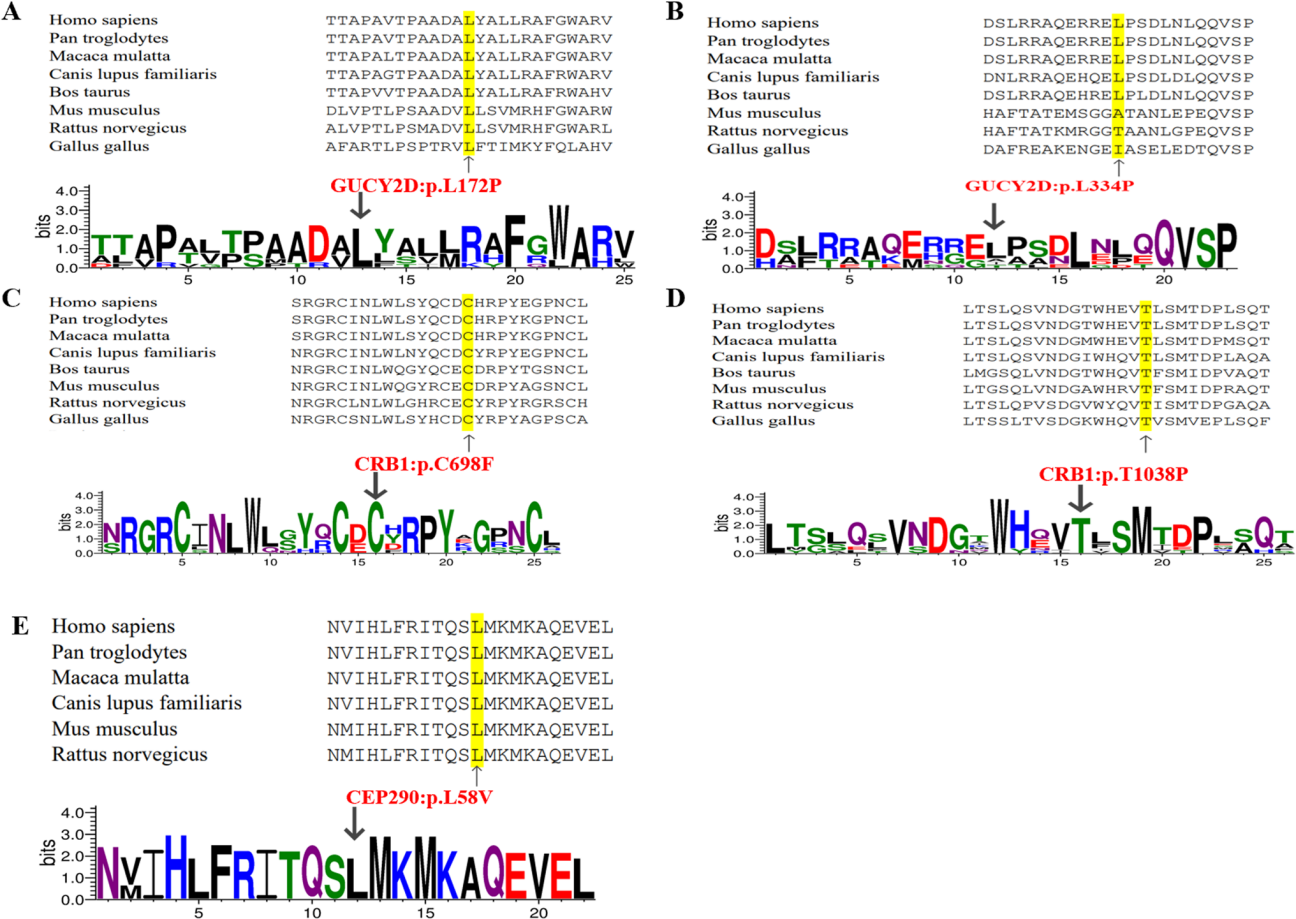
The homology analysis of the proteins from five novel missense variants. (**A**–**E**) Multiple alignments of amino acids showed that leucine at position 172, 334 of GUCY2D, cysteine at position 698 of CRB1, threonine at position 1038 of CRB1, and leucine at position 58 of CEP290 were highly conserved among different species

**Fig. 3:**
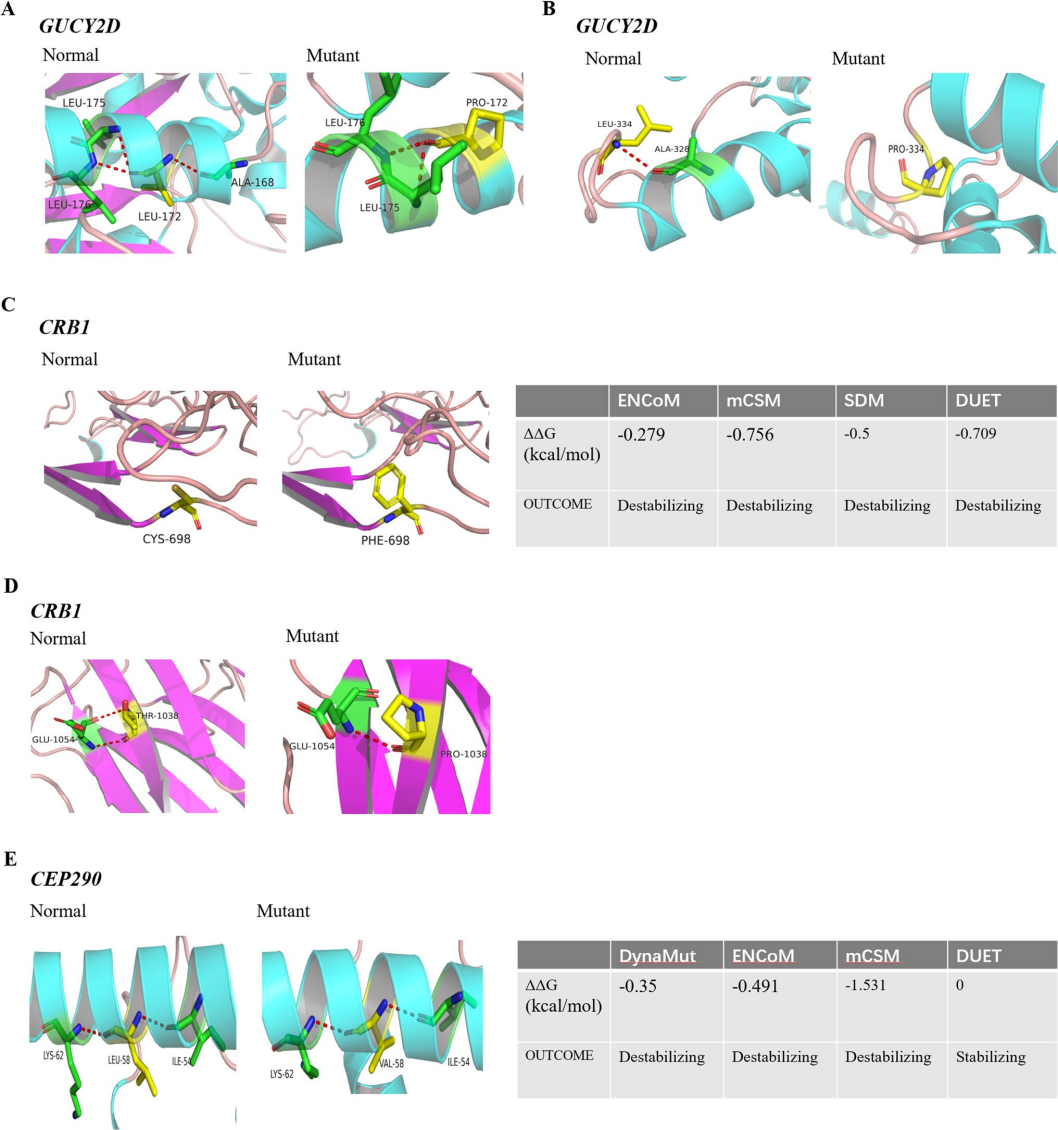
Three-dimension model construction for novel missense variations. The red dashed lines represent hydrogen bonds. (**A**) The model showed that a wild-type Leucine in GUCY2D was replaced by Proline at codon 172, which would make the connective hydrogen bands lost between the Leucine and Alanine at codon 168. (**B**) A wild-type Alanine in GUCY2D was replaced by Proline at codon 334. (**C**) A wild-type polar amino acid of Cysteine in CRB1 was replaced by a nonpolar amino acid of Phenylalanine at codon 698. Different programs predicted the Gibbs free energy (ΔΔ*G*) and showed the protein was unstable. (**D**) A wild-type polar amino acid of Threonine in CRB1 was replaced by a nonpolar amino acid of an amino acid of Proline at codon 1038, disrupting the hydrogen bonding. (**E**) A large-size wild-type Leucine was replaced by a small-size Valine at codon 58 in CEP290 ΔΔ*G* predicting the protein became unstable. All the above-mentioned amino acid substitutions may damage the stability of the protein structure and function

The novel missense variants identified in this study exhibit a high degree of conservation among several species, including *Homo sapiens*, *Pan troglodytes*, *Macaca mulatta*, *Canis lupus familiaris*, *Mus musculus*, and *Rattus norvegicus* (Fig. 2A–E). The missense variants of c.515 T > C (p.L172P) and c.1001 T > C (p.L334P) in *GUCY2D* have a common feature of a wild-type aliphatic Leucine replaced by a small size Proline, which are predicted to destruct the protein stabilization due to loss of the hydrogen bonds connected Leucine with other amino acids (Fig. 3A–B). A small-size wild-type Cystine replaced by a large-size mutant-type Phenylalanine in c.2093G > T (p.C698F) of *CRB1* would be predicted to destroy the structural stabilization (Fig. 3C). On the contrary, a large-size wild-type Leucine replaced by a small-size Valine in c.172C > G (p.L58V) of *CEP290* would also destroy the stabilization of protein (Fig. 3E), while the missense variant of c.3112A > G (p.T1038P) in *CRB1* would result in instability of the protein due to the loss of the hydrogen bonds connected Threonine with other amino acids (Fig. 3D).

### Diameters of vessels and blood flow

**Fig. 4:**
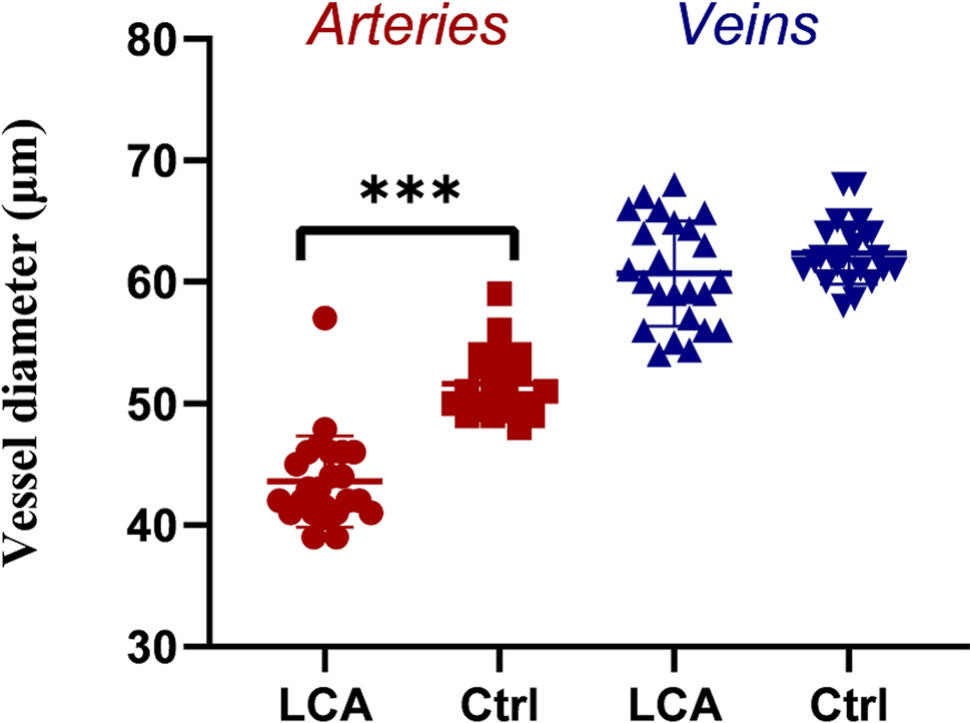
Scatter diagram of vessel diameters of LCA patients (LCA) and controls (Ctrl). ***P < 0.001

Twelve cooperative children (Supplement Table 5) between the ages of 3 and 14 (averaged 7.6 ± 3.5 years) were evaluated for retinal blood supply. Additionally, a control group of 12 individuals, matched for age (mean age 7.6 ± 3.5 years) and sex (three females and nine males), were included in the study. The average diameter of retinal arteries in LCA patients was calculated to be 43.6 ± 3.8 μm, while in the normal controls, it was 51.7 ± 2.6 μm. The average diameter of retinal veins in LCA patients was measured to be 60.7 ± 4.3 μm, whereas in the normal controls, it was 62.4 ± 2.5 μm. The retinal arteries were narrowed significantly in the LCA patients than in the normal controls (*P* < 0.001). However, the average diameter of retinal veins was not different significantly between the patient and controls (*P* = 0.159) (Fig. 4).

An average peak systolic velocity (PSV) of 16.3 ± 5.4 cm/s was observed in the ophthalmic artery (OA) of the patients, while the normal controls exhibited a PSV of 23.5 ± 6.4 cm/s. The PSV in the OA was found to be significantly decreased in the LCA eyes compared to the normal eyes (*P* = 0.0132). The OA showed an average pulsatility (PI) value of 3.6 ± 1.3 patients and 2.5 ± 1.1 in the normal controls. The PI in the OA was slightly increased in the patients than in the controls (*P* = 0.0488). However, the other two parameters, EDV and RI, did not exhibit significant differences between the patients and the normal controls in the OA. In addition, all four hemodynamic parameters were not found to have a difference between the patients and the normal controls in the CRA, PCA (Table 2), CA, ICA, and ECA (Supplement Table 6).

**Table 2:** Hemodynamic parameters in the ophthalmic artery, central retinal artery, and posterior ciliary artery in patients and normal control subjects

	Ophthalmic artery				Central retinal artery				Posterior ciliary artery			
	PSV (cm/s)	EDV (cm/s)	PI	RI	PSV (cm/s)	EDV (cm/s)	PI	RI	PSV (cm/s)	EDV (cm/s)	PI	RI
LCA	16.33 ± 1.43	2.38 ± 2.16	3.68 ± 1.33	0.84 ± 0.15	9.36 ± 4.41	2.21 ± 1.88	1.76 ± 0.41	0.78 ± 0.11	10.33 ± 4.10	1.83 ± 1.14	1.99 ± 0.62	0.81 ± 0.11
Control	23.53 ± 2.40	1.79 ± 1.28	2.52 ± 1.11	0.91 ± 0.07	10.03 ± 1.48	1.84 ± 0.86	1.90 ± 0.46	0.81 ± 0.08	9.36 ± 1.93	2.40 ± 1.08	1.56 ± 0.44	0.73 ± 0.13
*P*	**0.01**	0.51	**0.04**	0.24	0.66	0.58	0.43	0.51	0.54	0.26	0.10	0.17

Bold indicates statistically significant differences (*P* < 0.05)

*PSV*, peak systolic velocity; *EDV*, end diastolic velocity; *PI*, pulsatility index; *RI*, resistance index

## Discussion

In this study, we present a comprehensive analysis of the clinical characteristics of 52 LCA children and found 75 variants in 10 LCA-associated genes. In addition, our findings indicate significant alterations in the vascular characteristics of the retina.

Although several variants are classified as “variants of unknown significance (VUS)” according to the American College of Medical Genetics and Genomics (ACMG) criteria, we utilized a range of bioinformatics tools, such as SIFT, Polyphen-2, PROVEAN, and Splicing AI analysis, the results from these tools suggested a likelihood of pathogenicity, with the 3D model of the protein predicting detrimental effects.

As a group of IRDs, the LCA is undoubtedly one of the earliest and most severe forms. In the clinical setting, young children, especially those of a very tender age, often present challenges in cooperating with comprehensive eye examinations to obtain reliable measures of vision. This difficulty can hinder ophthalmologists in making an accurate diagnosis of LCA in early childhood. For example, a 14-year-old affected boy in our cohort was treated for “amblyopia” over 10 years, as he was diagnosed with nystagmus, hyperopia, and amblyopia. He was eventually identified as LCA1 based on his ERG recordings and genetic tests. In our experience, ERG examination and genetic testing help to assess unexplained low vision and nystagmus in patients, especially in LCA patients. The average age of patients in our cohort was relatively young, with an average visit age of 4.1 ± 3.1 years and an average onset age of 4.4 ± 2.5 months, and some clinical features of LCA such as photophobia, nyctalopia, and keratoconus were not evident at their visit time. Instead, nystagmus, poor fixation, and poor smooth pursuit were their main symptoms. In the evaluation of the fundus, we found that a common feature was attenuation of the retinal vessels, although the appearance of the fundus varied from patient to patient. To objectively evaluate the anatomical changes in the retinal vessels, AI techniques were employed to compare the diameter of the vessels between the patients and the normal controls and found that the retinal arteries in the LCA patients were indeed narrowed. Previous studies showed that the visual field loss was correlated with attenuation of the retinal arteries and veins in retinitis pigmentosa (RP) which was characterized by progressive visual field loss due to photoreceptor cell degeneration [9]. Vascular densities were found to be lower in RP, Stargardt disease (STGD), and cone-rod dystrophy (CRD) [10]. The anatomical and functional changes in the retinal vessels in patients with RP have been suggested to be caused by loss of function in the outer retinal region, which may reduce the metabolic demands on the retina due to decreased trophic factors [11, 12].

In addition to observed attenuation of retinal vessels, alterations in hemodynamic parameters in OA were identified in patients with LCA, specifically a decrease in PSV and an increase in PI. These changes are anticipated to contribute to diminished retinal blood flow. Hemodynamic parameters change accordingly with the narrowing of the vessels in LCA patients and may serve as a potential indicator for monitoring disease progression and even predicting prognosis. Similar findings have been reported in patients with retinitis pigmentosa, where both PSV and EDV of the CRA decrease compared to normal controls [9].

Previous studies have shown that common mutations have been detected in the *GUCY2D*, *RPE65*, *CRB1*, *CEP290*, and *RDH12* genes, occurring at frequencies of 10% or more [2]. However, in our cohort, the leading cause of LCA is the *RPGRIP1* mutations which account for 26.9% (14/52) of our patients, followed by *CEP290* (19.2%, 10/52), and *GUCY2D* (15.4%, 8/52). It has been suggested that frequency is related to ethnic background. Mutations in *CEP290*, *GUCY2D*, and *RPE65* are generally more frequent in Caucasian populations [13], while mutations in *CRB1*, which accounts for 13.6% of LCA cases, are believed to be the leading causative genetic mutation in the Chinese cohort, followed by *GUCY2D* [14–18]. However, the leading causative mutation observed in our cohort was *RPGRIP1* followed by *CEP290*, illustrating the difference between the European and Chinese populations. In addition, even within the Chinese population, the frequency of gene mutations may vary due to factors such as the sample size and source, for example, only four patients in our cohort were identified to carry the *CRB1* variants.

Previous studies have shown that myopia is associated with the *RPE65* mutation [19]. However, in our cohort, all four patients with *RPE65* variants had hyperopia, and in contrast, two of 14 patients with *RPGRIP1* variants had myopia. RPGRIP1 is expressed richly in connecting cilia in retinal photoreceptors, which is an important conduit through which the nascent proteins must be transported to the outer segments of the photoreceptor cells to satisfy their daily metabolic needs [20]. RPGRIP1 usually interacts with the RPGR via its carboxy-terminal portion [21]. *RPGR* is located on the X chromosome. Mutations in *RPGR* can lead to X-linked retinitis pigmentosa and myopia [22]. It is suggested that the critical site for myopia in photoreceptors appears to be the transport area between the inner and outer segments [23]. Thus, we hypothesize that, like patients with *RPGR* mutation, patients with *RPGRIP1* may be more prone to developing myopia.

There are some limitations to this study. First of all, we found that PSV was decreased in the ophthalmic artery, but was not changed in the central retinal vascular compared with the normal control, which may be due to the small sample size and a certain deviation in the comparison of the differences in the parameters of the terminal small vessels. In addition, we lack sufficient evidence to support that patients with *RPGRIP1* mutations are more prone to developing myopia than those with *RPE65*. Further follow-up is needed to prove our hypothesis.

In summary, we investigated the clinical and genetic characteristics of 52 LCA patients in this study. We found that early screening of hemodynamic parameters may be helpful to monitor disease progression and understand IRD-related vascular abnormalities in future studies. In addition, we identified 29 novel variants in 10 LCA-associated genes and found that mutations in *RPGRIP1* may be the leading cause of LCA in Chinese patients. The novel-identified mutations will expand the mutation spectrum of disease-causing genes.

## Supplementary Information

Below is the link to the electronic supplementary material.

Supplementary file1 (DOCX 2494 KB)

Supplementary file2 (DOCX 67 KB)
