# Supplementary material for: Clinical and genetic studies for a cohort of patients with Leber congenital amaurosis
Source: Graefes Arch Clin Exp Ophthalmol. 2024 Apr 25;262(9):3029–38. doi: 10.1007/s00417-024-06450-9 (PMC11377616; doi:10.1007/s00417-024-06450-9)

**Supplement Figure 1.** The figure illustrates AI-based measurement of retinal artery diameters, emphasizing the identification and segmentation process within the retinal structure. The red color represents arteries, while the blue color represents veins. The diameters obtained are the averages for all blood vessels


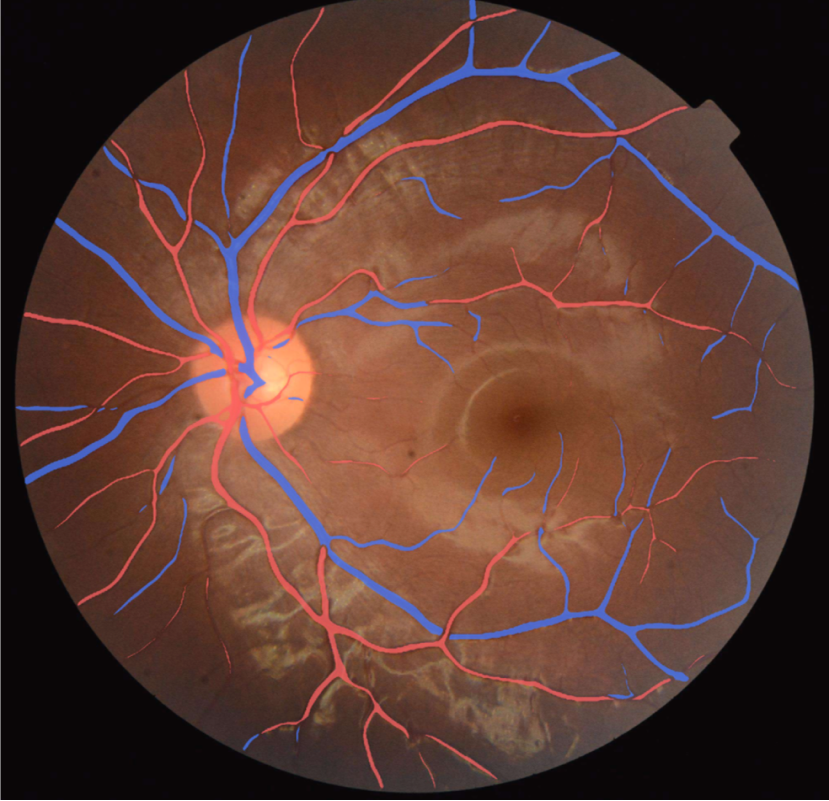


**Supplement Figure 2**. Summary of the proportion of patients in this study with mutations of the involved genes and type.


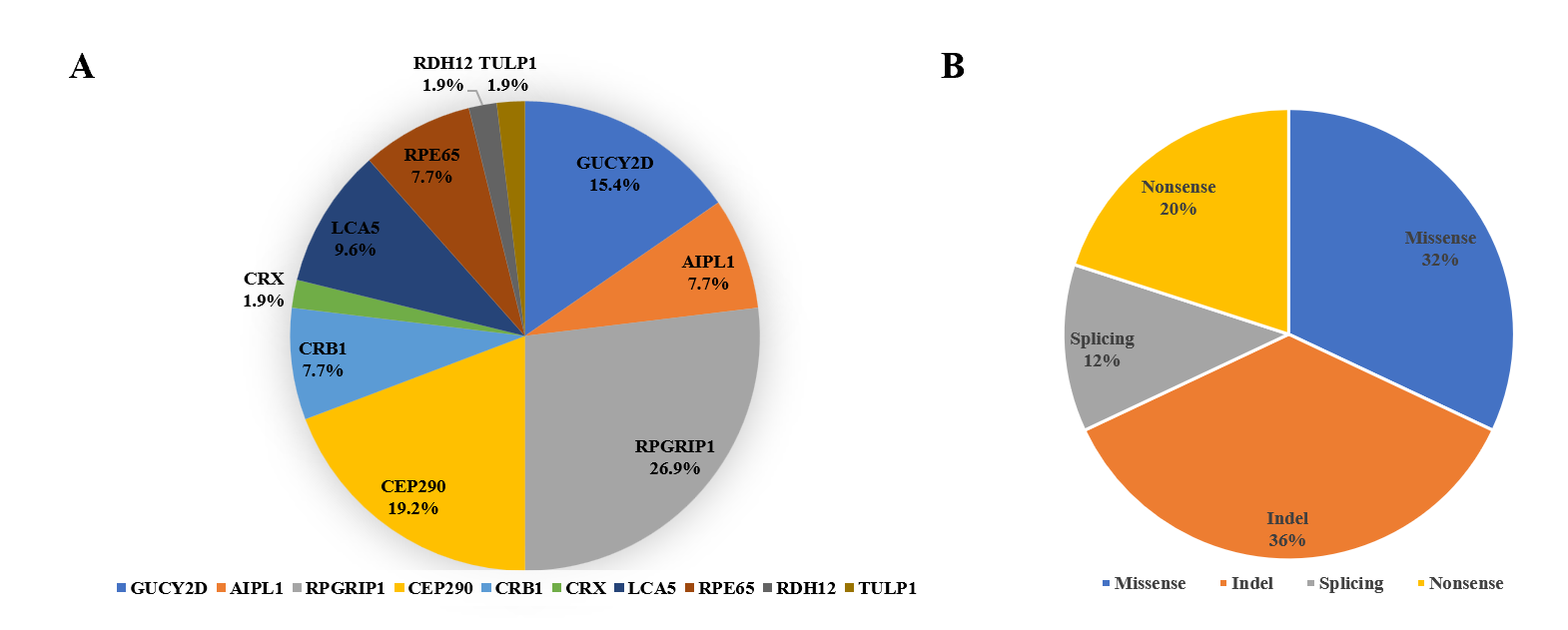


**Supplement Figure 3.** Pedigrees of the families with novel variants in LCA genes. Filled symbols indicate individuals affected with LCA. Arrows indicate probands. Chromatograms showing novel variants identified in LCA genes.


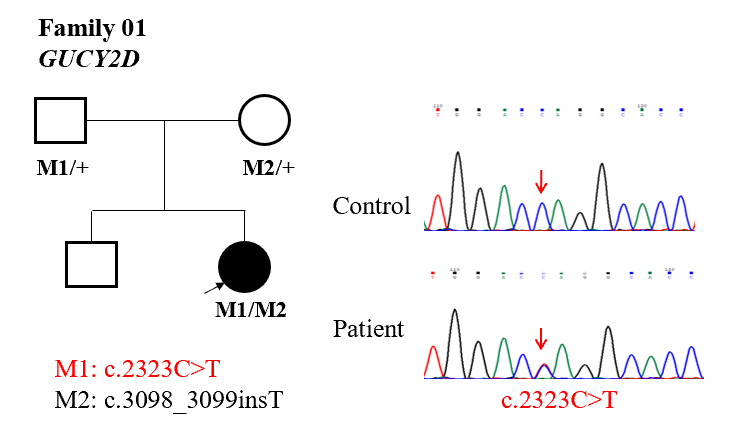

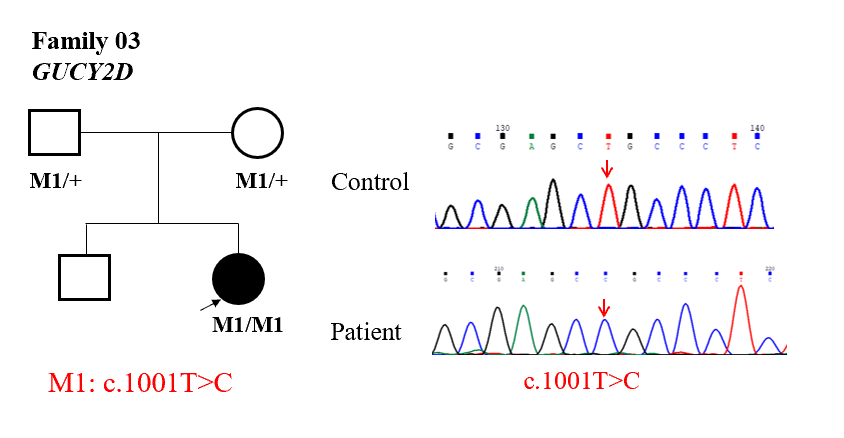


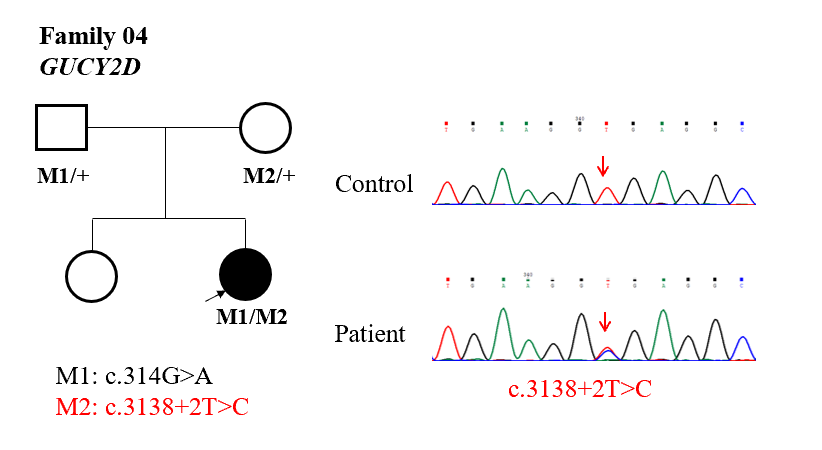


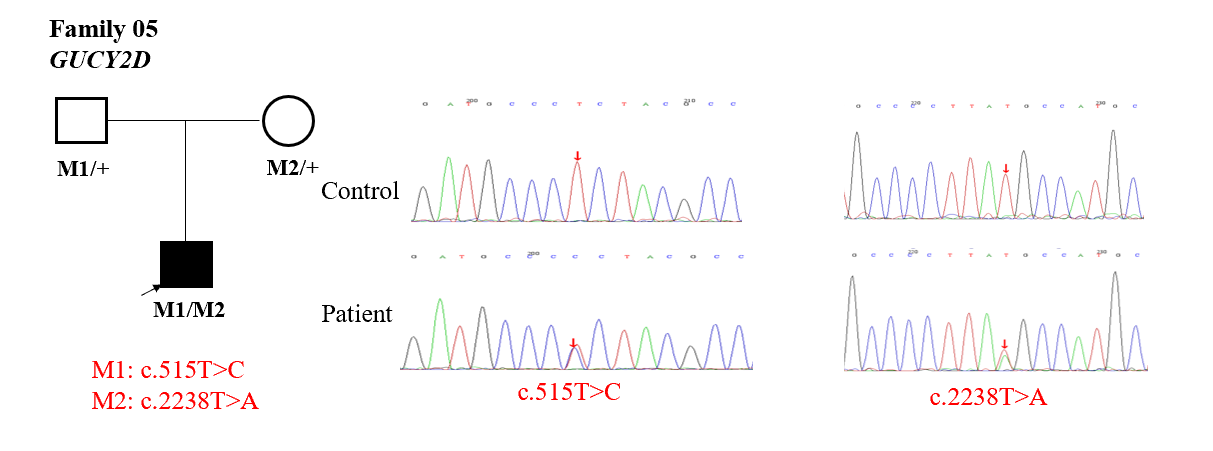


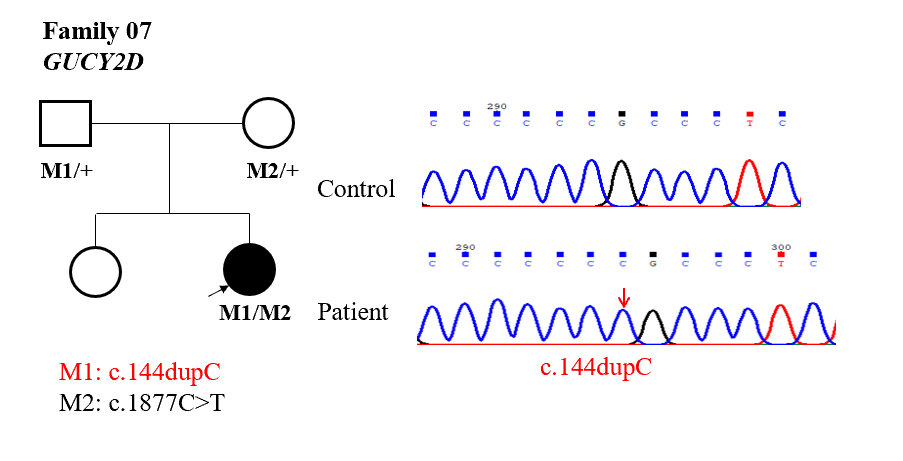

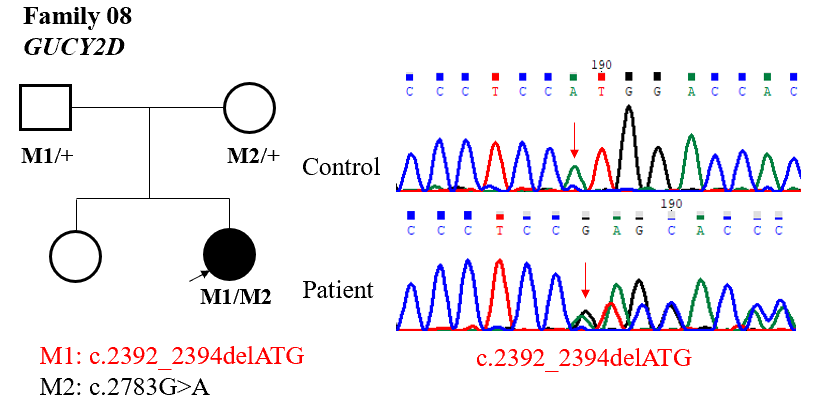


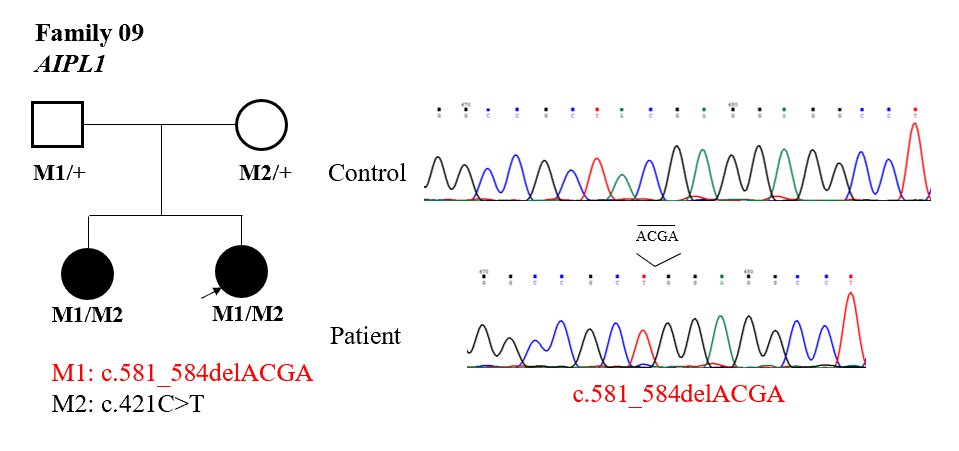

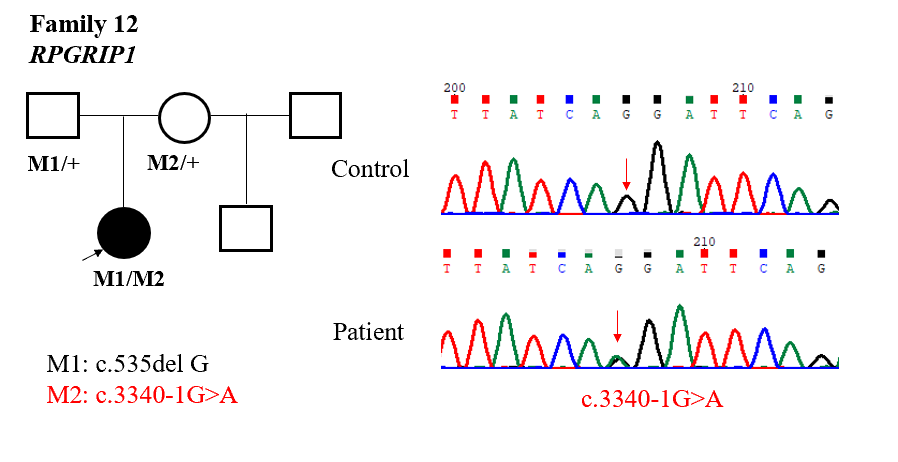


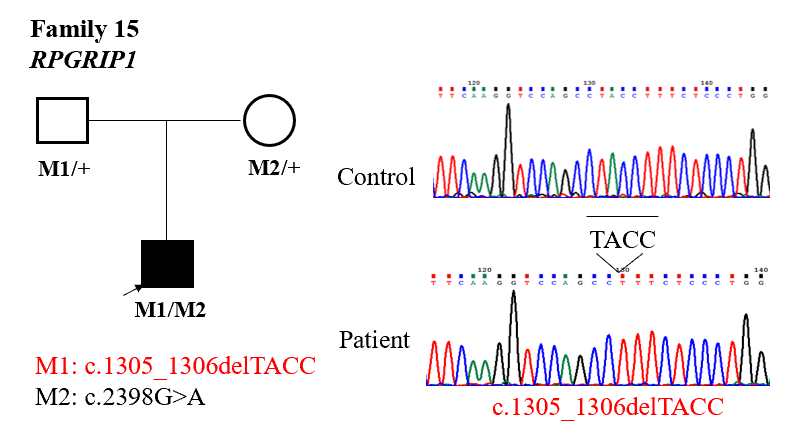

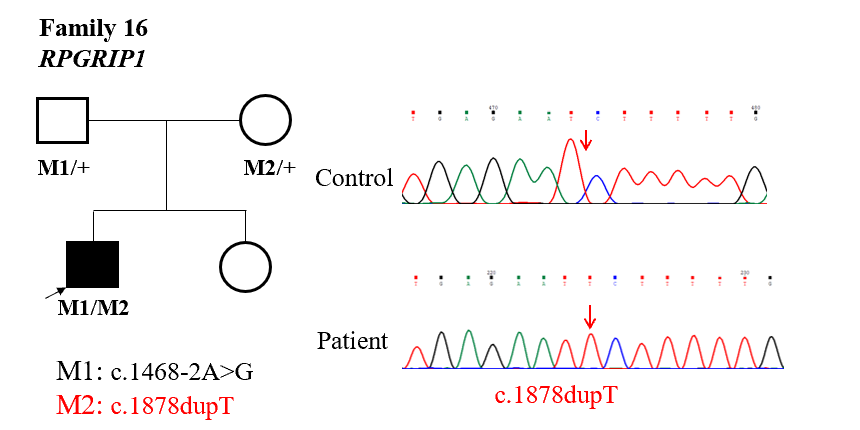


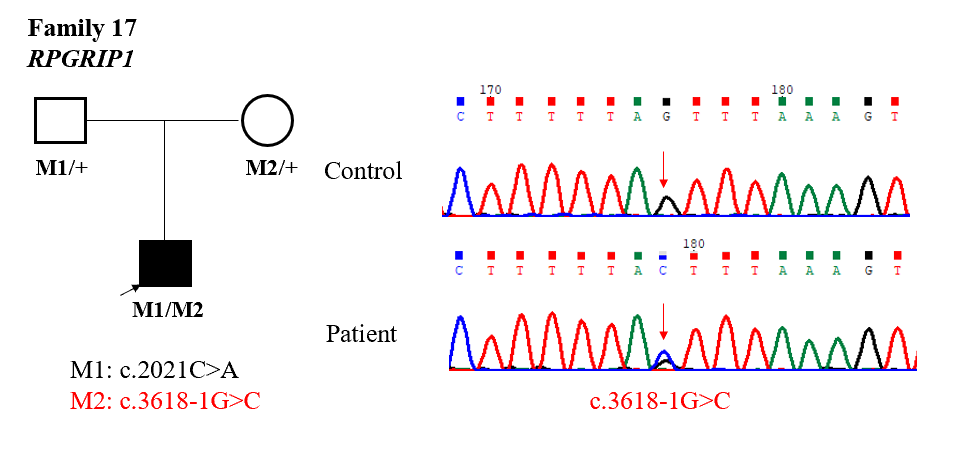

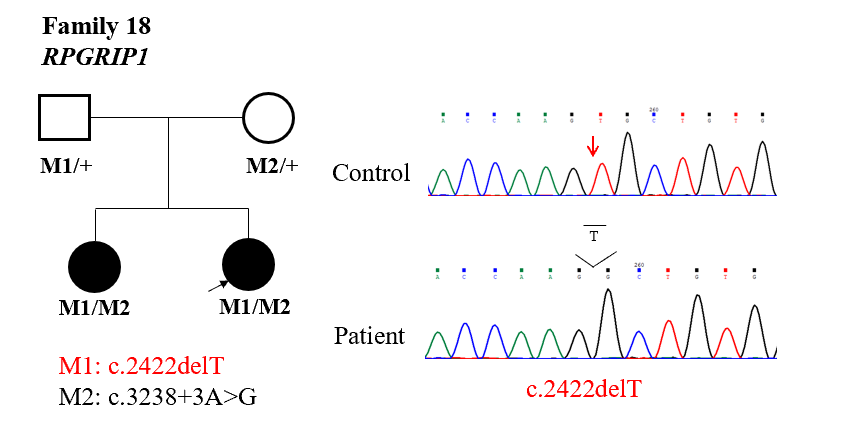


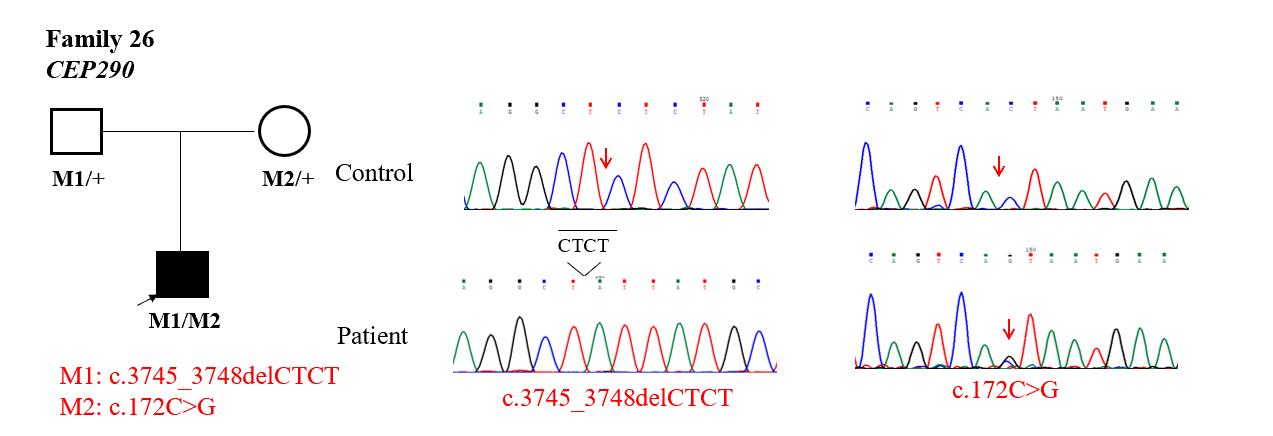


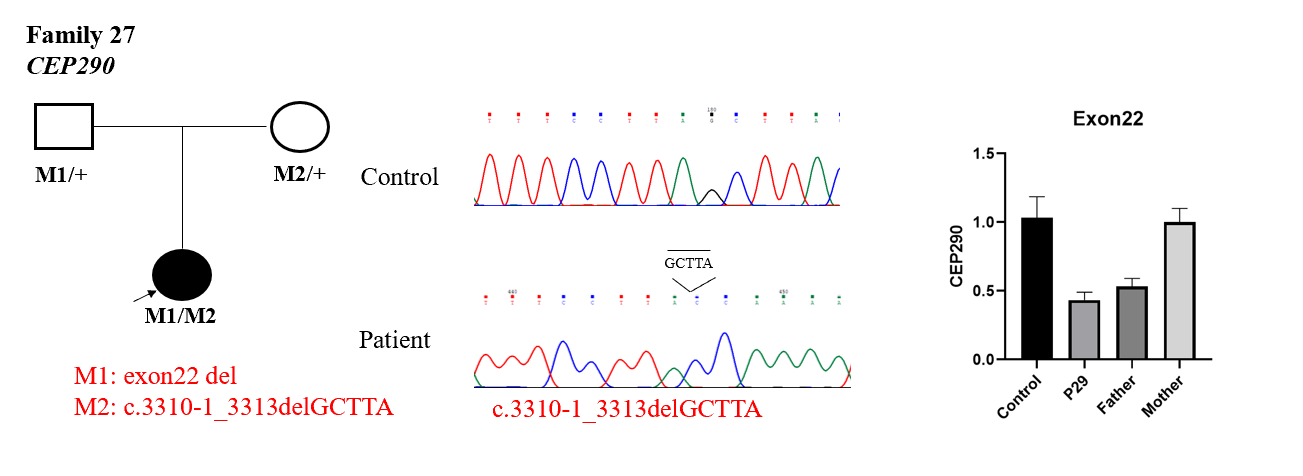


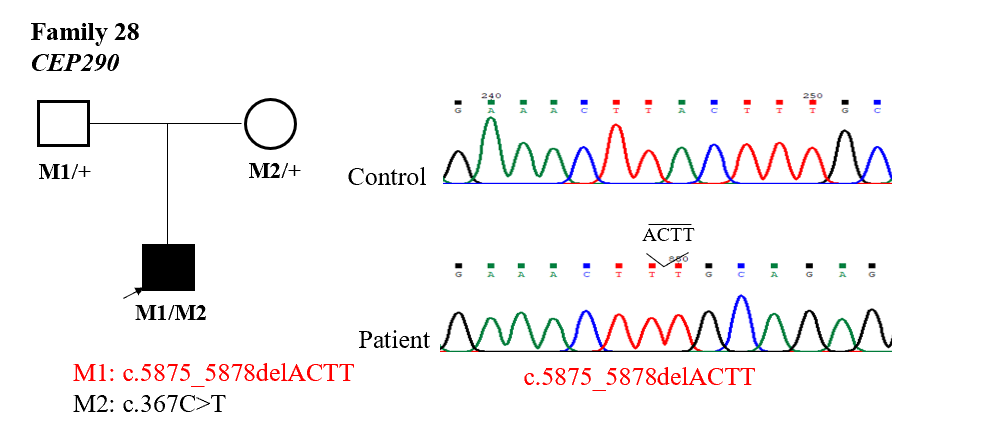

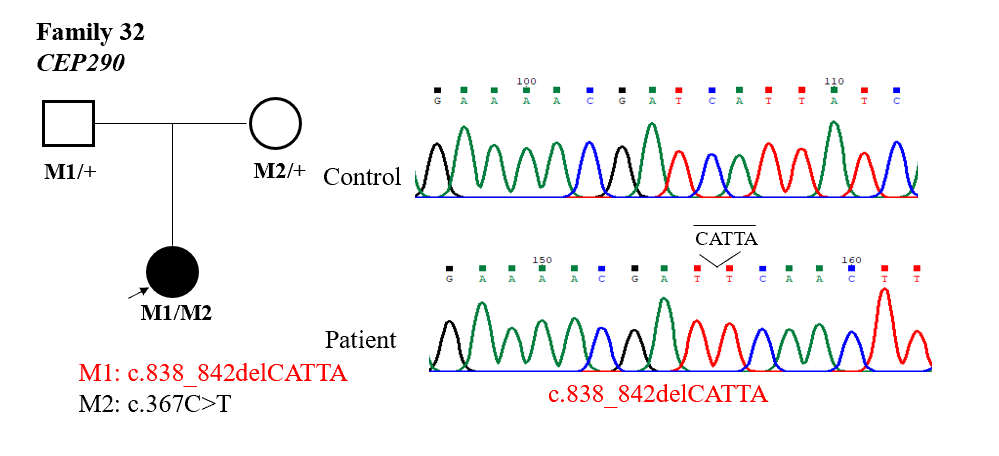


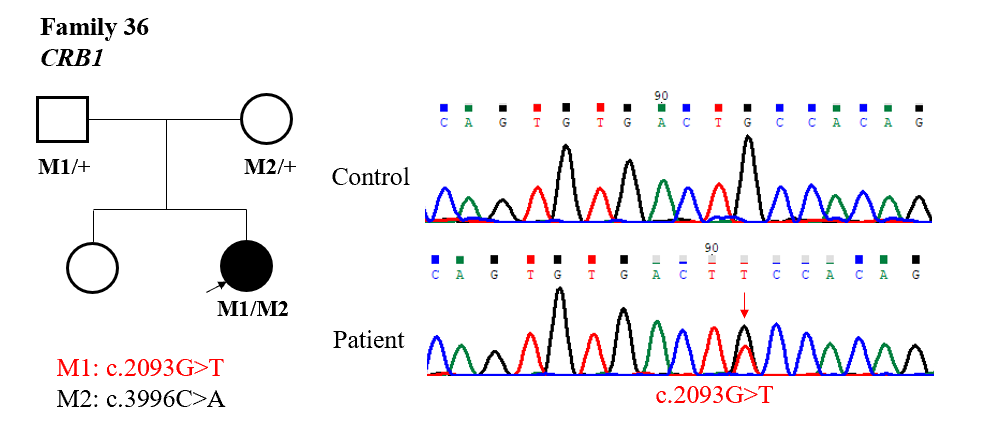

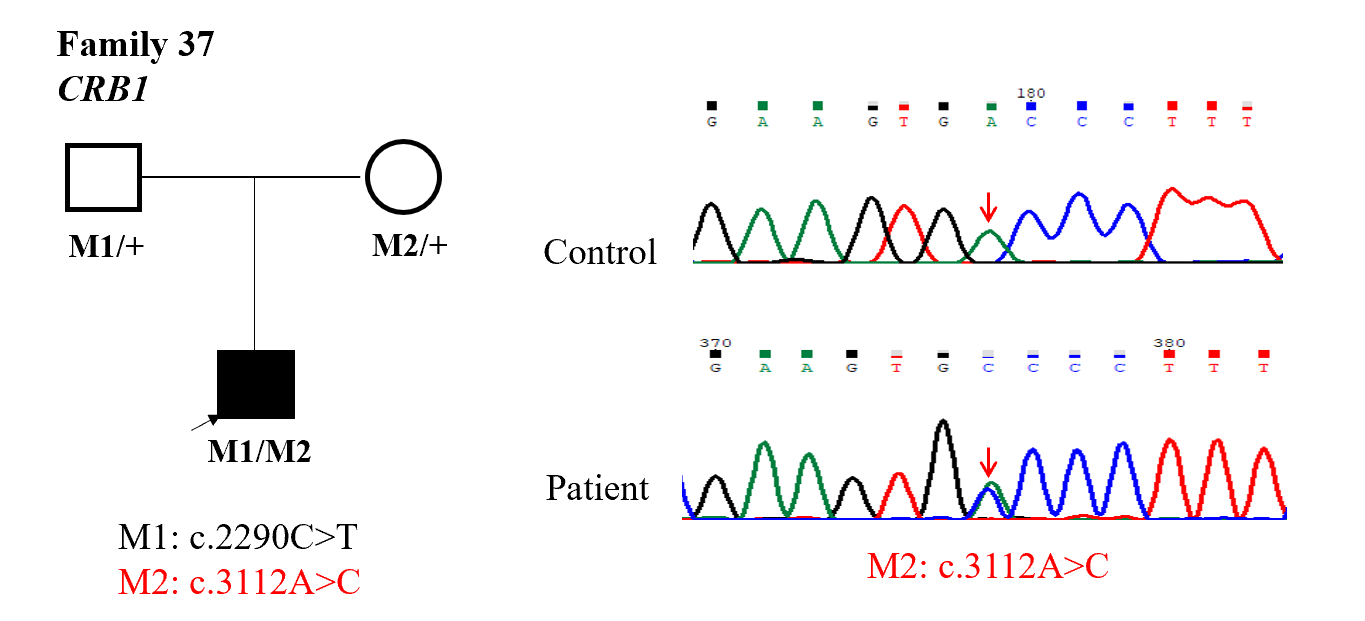


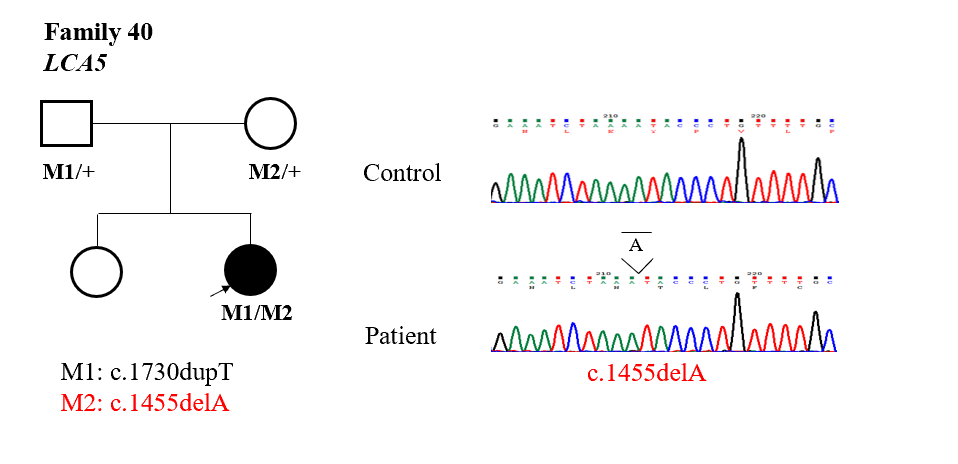

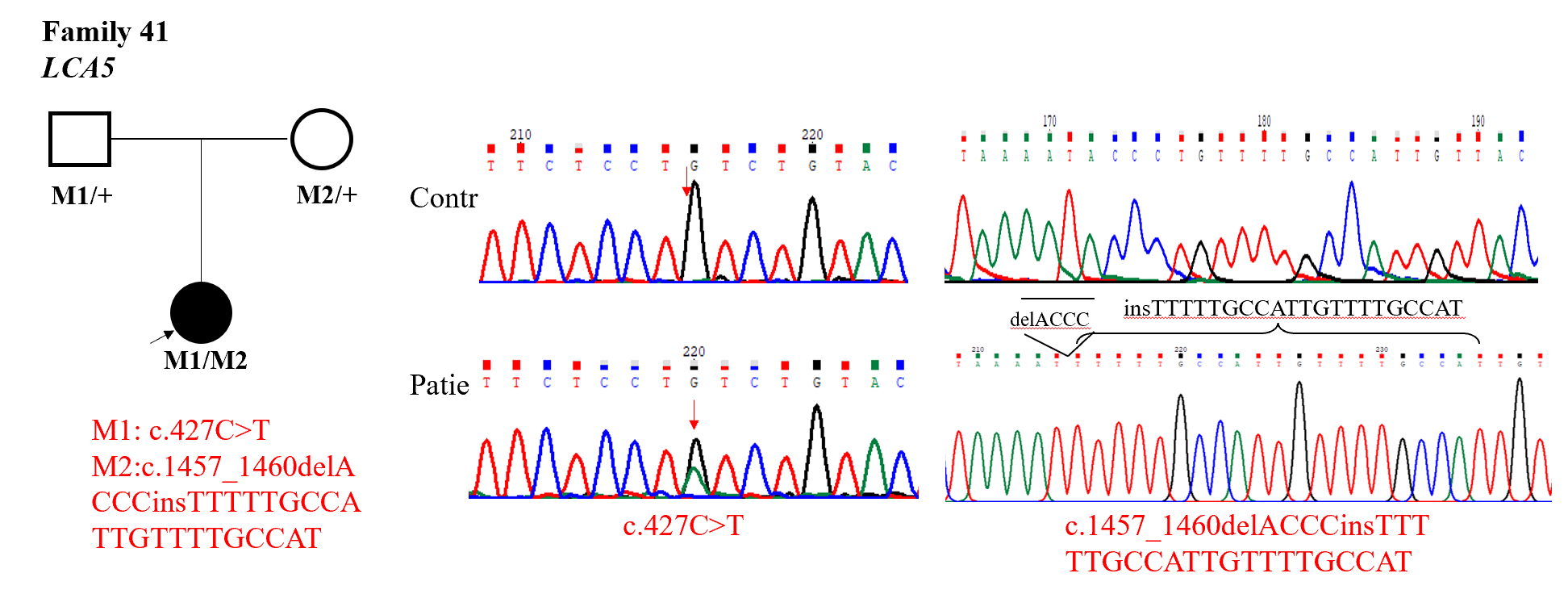

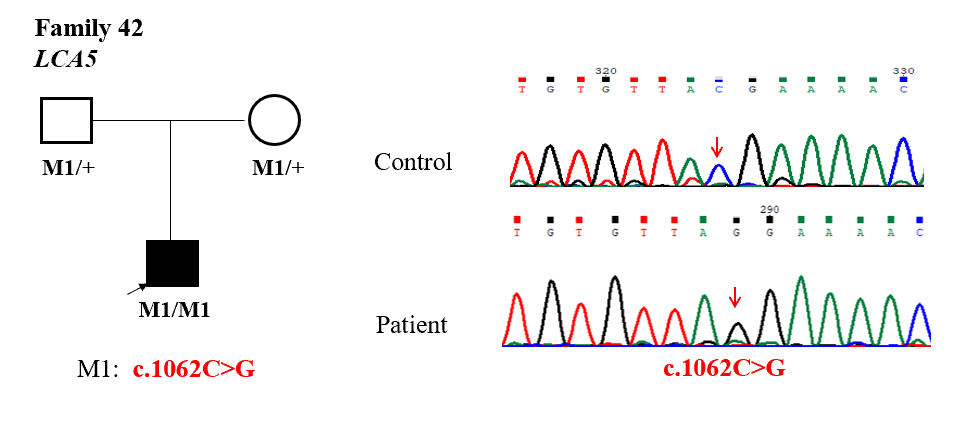

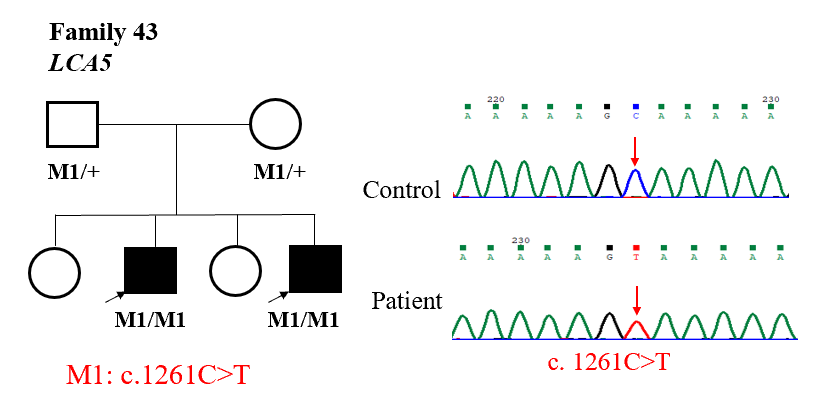


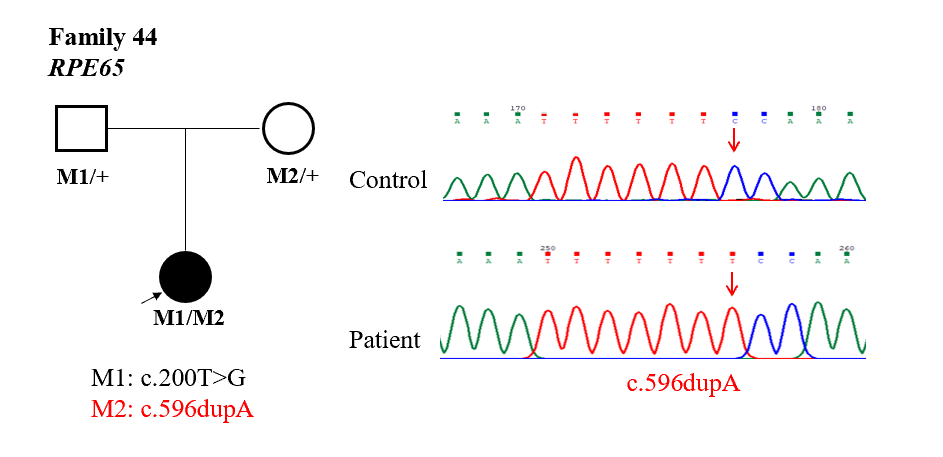

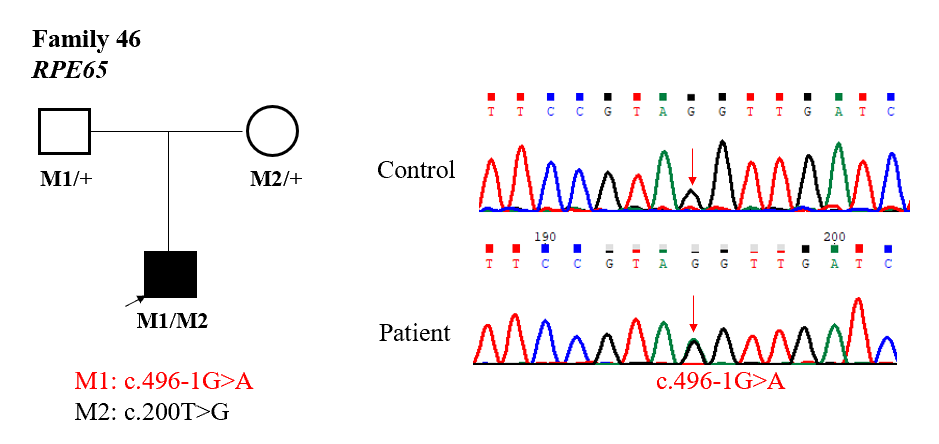


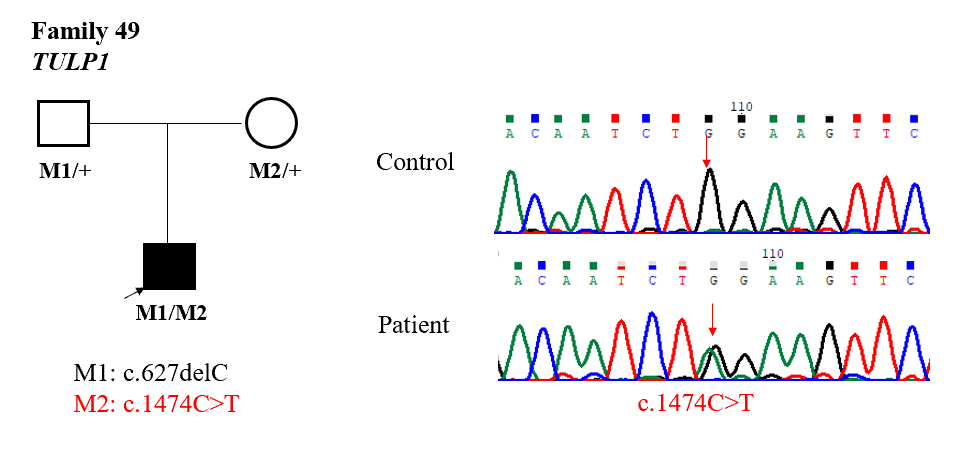

Supplement: Supplementary file 1 — Supplementary file1 (DOCX 2494 KB) [file 417_2024_6450_MOESM1_ESM.docx]
