# Supplementary material for: Clinical and genetic studies for a cohort of patients with Leber congenital amaurosis
Source: Graefes Arch Clin Exp Ophthalmol. 2024 Apr 25;262(9):3029–38. doi: 10.1007/s00417-024-06450-9 (PMC11377616; doi:10.1007/s00417-024-06450-9)
Supplement: Supplementary file 2 — Supplementary file2 (DOCX 67 KB) [file 417_2024_6450_MOESM2_ESM.docx]

| **Gene** | **Primer name** | **Primer sequence** |
| --- | --- | --- |
| *GUCY2D* | Exon2F  Exon2R | CTTGGAGAAACTCGGGGTTA  AGAGAAGATGGGGTCGCAAG |
| *AIPL1* | Exon4F  Exon4R | TATGCACTTGACCAGCAAGC  AGGGAGAAGGTCAGCCATGA |
|  | Exon10F  Exon10R | TGGGGTATCCATCTGAGAGC  GCTCTTCAAGGTCCAGCCTA |
|  | Exon14F  Exon14R | GGCAGCTTTCTTTCCCCTCT  TGGCAGGAATAACAACATGG |
|  | Exon16F  Exon16R | AAGCTGCCCTTTTCCTCAAT  AGGGCGTGAAGATGTAATAACC |
| *CEP290* | Exon9F  Exon9R | CAGGAAATGGAGAAGATGACTG  GTGTTTGTGAGGTGATTGGAGA |
|  | Exon29F  Exon29R | AGGCCAAGTAAAGAGGATTGC  GCAAATCTTGAGAGGTCACTTCC |
|  | Exon31F  Exon31R | AAGTCGCTCATTGCCAAGTT  GGGCTCCTTTGGTATCCTTT |
|  | Exon43F  Exon43R | TGGATTTTCCTGTGATCTCG  CCAAAGCTCGTATTCCCAAA |
| *LCA5* | Exon9F  Exon9R | CAGAGTGGGAAACTGGAAGG  CCTGTATGTTTTGGGGCTTC |
| *RPE65* | Exon6F  Exon6R | CACCCCCACATTGAAAATGA  GGATGAGGGCAGTACTTCTGA |

Supplement Table 1

PCR Primer：

Supplement Table 2

qPCR Primer：

| **Gene** | **Primer name** | **Primer sequence** |
| --- | --- | --- |
| *CEP290* | Exon22F  Exon22R | TGGCAGGGCATTCACATATC  ATCTGCATGCTTTGGTGATG |
| *RPGRIP1* | Exon1F  Exon1R | TCAGTGTCCTCTGGGATCTCTT  TCTGTGAAGGCCAGCAAGAT |
|  | Exon23F  Exon23R | AATGGAGGCAAGGGAAAAGT  TGGGCATCAGCTGCCTATAC |
| *GAPDH* | F  R | TATGGTCCTGTCCCCATCTC  CAGCAGAGAAGCAGACAGTTATG |

Supplement Table 3 Clinical symptoms and examinations in 52 children with Leber congenital amaurosis

| Patient number | Gene | Sex | Onset Symptoms | Age at latest examination | Ophthalmological Symptoms | Refraction  (OD/OS) | BCVA  (OD/OS) | Funds examination | ERG |
| --- | --- | --- | --- | --- | --- | --- | --- | --- | --- |
| 01 | *GUCY2D* | F | 3m-poor pursuit | 5m | Nystagmus, photophobia, ODS | NA | NA | NA | Extinguished |
| 02 | *GUCY2D* | M | 6m-poor pursuit | 2y | Nystagmus, ODS | +7.00 DS/+1.50 DC×130  +5.00 DS/+1.50 DC×50 | NA | AV | Severely diminished |
| 03 | *GUCY2D* | F | 3m-poor pursuit | 1y | Nystagmus, photophobia, cataract | +5.00 DS/+1.00 DC×80  +5.25 DS/+1.00DC×90 | NA | AV | Extinguished |
| 04 | *GUCY2D* | F | 2m-nystagmus | 6y | Photophobia, SPR | +4.75 DS/+2.50 DC×95  +4.75 DS/+2.00 DC×95 | 20/500  20/500 | AV | Extinguished |
| 05 | *GUCY2D* | M | 3m-nystagmus | 8m | ODS | +8.00DS  +7.00DS | NA | NA | Extinguished |
| 06 | *GUCY2D* | F | 4m-nystagmus | 3y | ODS, SPR, esotropia | +5.25 DS/+2.50 DC×80  +5.25 DS/+1.00 DC×100 | CF  CF | AV | Extinguished |
| 07 | *GUCY2D* | F | 3m- ODS | 3m | Photophobia, no fixation | NA | NA | NA | Extinguished |
| 08 | *GUCY2D* | M | 3m-nystagmus | 14y | Photophobia, ODS, deep-set eyeballs, cataract | +5.25 DS/+1.50 DC×20  +4.75 DS/+1.50 DC×80 | 20/400  20/400 | AV | Extinguished |
| 09 | *AIPL1* | F | 6m-poor pursuit, ODS | 3y | Nystagmus, photophobia, low-set eyeballs | +5.00DS/+2.50 DC×95  +4.50DS/+2.00 DC×50 | LP  LP | AV, BMF | Extinguished |
| 10 | *AIPL1* | F | 8m-poor pursuit | 5y | Photophobia | +4.00DS/+1.50 DC×95  +4.75DS/+1.50 DC×80 | CF  CF | BMF | Extinguished |
| 11 | *AIPL1* | M | 5m-nystagmus | 1y | ODS, SPR | NA | NA | NA | Extinguished |
| 12 | *AIPL1* | F | 3m-nystagmus | 2y | ODS | +5.00 DS/-1.50 DC×150  +4.75 DS/-1.50 DC×20 | LP  LP | Peripheral pigmentary retinopathy | Extinguished |
| 13 | *RPGRIP1* | F | 5m-nystagmus, poor pursuit | 14y | Photophobia, SPR | -2.75 DS/+3.50 DC×95  -1.75 DS/+3.50 DC×80 | 20/300  20/300 | Grey optic disc, AV, retinal exudates, RPD | Extinguished |
| 14 | *RPGRIP1* | M | 3m-nystagmus, poor pursuit | 3y | SPR, no fixation, ODS | -3.00 DS/+2.75 DC×120  -4.00 DS/+3.00 DC×60 | LP  LP | AV, BMF | Extinguished |
| 15 | *RPGRIP1* | F | 6m- ODS | 3y | Nystagmus, poor pursuit | +6.00 DS/-1.50 DC×180  +5.50 DS/-1.50 DC×180 | LP  LP | RPE atrophy, RPD | Extinguished |
| 16 | *RPGRIP1* | M | 5m-poor pursuit | 2y | Nystagmus, esotropia | +3.50 DS+1.00 DC×100  +3.50 DS+1.25 DC×90 | NA | BMF | Extinguished |
| 17 | *RPGRIP1* | M | 2m-nystagmus | 10y | Photophobia | +2.50 DS+0.50 DC×95  +2.50 DS+1.00 DC×95 | 20/1000  20/1000 | RPD | Extinguished |
| 18 | *RPGRIP1* | M | 10m- poor pursuit | 6y | Photophobia, nystagmus | +3.00 DS/+2.00 DC×100  +3.25 DS/+2.50 DC×80 | 20/250  20/250 | AV, RPD | Extinguished |
| 19 | *RPGRIP1* | F | 2m-nystagmus | 6y | SPR | +5.00 DS  +4.50 DS | 20/1000  20/400 | BMF | Extinguished |
| 20 | *RPGRIP1* | F | 1m-nystagmus | 3y | SPR | +3.25 DS/-2.00 DC×180  +5.50 DS/-2.00DC×20 | LP  LP | RPD | Extinguished |
| 21 | *RPGRIP1* | M | 3m-nystagmus | 4y | Photophobia | +2.50 DS/+4.00 DC×80  +2.50 DS/+4.25 DC×100 | 20/200  20/200 | AV | Extinguished |
| 22 | *RPGRIP1* | M | 5m-poor pursuit | 3y | Photophobia, nystagmus, SPR | +3.25 DS/+1.00 DC×20  +3.50 DS/+0.50 DC×150 | LP  LP | AV | Extinguished |
| 23 | *RPGRIP1* | F | 1m- nystagmus | 5m | Poor pursuit, SPR | NA | NA | AV | Extinguished |
| 24 | *RPGRIP1* | M | 4m-nystagmus | 6y | Esotropia | -1.50 DS/+3.75 DC×95  -1.25 DS/+4.50 DC×80 | 20/125  20/125 | BMF | Extinguished |
| 25 | *RPGRIP1* | F | 5m-poor pursuit | 5y | Nystagmus, ODS, SPR | +0.50 DS/+2.75 DC×100  +1.25 DS/+2.00 DC×70 | LP  LP | AV | Extinguished |
| 26 | *RPGRIP1* | M | 6m-nystagmus | 9m | Nystagmus, ODS, SPR | +4.00 DS/+1.00 DC×90  +5.50 DS/+1.00 DC×90 | NA | AV | Extinguished |
| 27 | *CEP290* | M | 2m-nystagmus | 1y | ODS, SPR | +8.00 DS  +9.00DS | NA | Patchy retinal degeneration | Extinguished |
| 28 | *CEP290* | M | 6m-nystagmus | 3y | Exotropia | +3.50 DS/+2.00 DC×20  +4.00 DS/+2.00 DC×80 | LP  LP | Salt and pepper funds | Extinguished |
| 29 | *CEP290* | F | 2m-nystagmus | 3y | Exotropia | +6.00 DS/+1.00 DC×100  +6.00 DS/+1.75 DC×100 | NA | Patchy retinal degeneration | Extinguished |
| 30 | *CEP290* | M | 3m-nystagmus | 2y | ODS | NA | NA | NA | Extinguished |
| 31 | *CEP290* | M | 3m-nystagmus, ODS | 3y | Photophobia | +5.00 DS/+1.50 DC×90  +5.25 DS/+1.00 DC×20 | LP  LP | AV | Extinguished |
| 32 | *CEP290* | M | 3m-poor pursuit | 7y | Nystagmus, SPR | +3.75 DS/+1.50 DC×100  +4.00 DS/+1.50 DC×90 | 20/200  20/200 | BMF | Extinguished |
| 33 | *CEP290* | M | 6m-poor pursuit, nystagmus | 3y | Photophobia, deep-set eyeballs | +8.50 DS/+1.25 DC×120  +7.00 DS/+1.50 DC×60 | LP  LP | BMF, AV | Extinguished |
| 34 | *CEP290* | M | 5m-poor pursuit | 5m | Nystagmus | +10.00 DS/+1.50DC×95  +9.25 DS/ | NA | Patchy retinal degeneration | Severely diminished |
| 35 | *CEP290* | M | 1m-nystagmus | 3y | ODS | +4.25 DS/+3.00 DC×90  +4.00 DS/+3.25 DC×85 | LP  LP | AV, BMF | Extinguished |
| 36 | *CEP290* | M | 1m-nystagmus | 3y | ODS, SPR | +8.00 DS/  +8.50 DS/-1.00 DC×180 | 20/250  20/250 | AV, salt and pepper funds | Extinguished |
| 37 | *CRB1* | M | 10m-nystagmus | 6y | SPR, low-set eyeballs | +4.50 DS/+2.00 DC×20  +5.00DS/+2.00 DC×180 | 20/200  20/200 | BMF | Severely diminished |
| 38 | *CRB1* | F | 6m-nystagmus | 3y | No fixation | +5.50 DS/+2.00DC×60  +5.00 DC/+2.00DC×120 | CF  CF | BMF | Extinguished |
| 39 | *CRB1* | M | 1y-nystagmus | 3y | SPR | +7.00 DS/+1.00DC×85  +8.00 DS/+1.25DC×75 | 20/500  20/500 | BMF, drusen of the optic nerve head | Extinguished |
| 40 | *CRB1* | M | 1y-nystagmus | 4y | Exotropia | +1.50 DS/+2.00 DC×95  +2.00 DS/+ 2.00DC×75 | 20/100  20/100 | RPD | Extinguished |
| 41 | *CRX* | M | 1m-poor pursuit | 1y | ODS | NA | NA | HP | Extinguished |
| 42 | *LCA5* | F | 6m-poor pursuit | 8y | Nystagmus, exotropia | +1.00 DS/+2.75 DC×85  +0.5 DS/+3.50 DC×95 | 20/200  20/200 | BMF | Extinguished |
| 43 | *LCA5* | F | 5m-poor pursuit | 3y | ODS, SPR | +3.00 DS/+1.75 DC×20  +3.50 DS/+1.75 DC×90 | LP  LP | AV, BMF | Extinguished |
| 44 | *LCA5* | M | 5m-nystagmus | 6y | SPR | +3.50 DS/+2.00 DC×110  +3.50 DS/+2.70 DC×75 | 20/100  20/100 | AV, BMF | Extinguished |
| 45 | *LCA5* | M | 4m-nystagmus, no fixation | 3y | SPR, exotropia | +6.50 DS/+0.75 DC×165  +7.50 DS/+0.75 DC×80 | NA | AV, BMF | Extinguished |
| 46 | *LCA5* | M | 5m-nystagmus, poor pursuit | 10y | Night blindness | +6.25 DS/+2.00 DC×115  +7.00 DS/+2.00 DC×65 | 20/100  20/200 | Macular degeneration | Extinguished |
| 47 | *RPE65* | F | 5m-nystagmus | 3y | SPR | +4.50 DS/+1.00 DC×70  +5.00 DS/+1.00 DC×100 | NA | AV | Extinguished |
| 48 | *RPE65* | M | 3m-poor pursuit | 7y | Nystagmus | +1.50 DS/+2.00 DC×95  +2.00 DS/+2.00 DC×75 | 20/200  20/200 | BMF | Extinguished |
| 49 | *RPE65* | M | 4m-nystagmus, no fixation | 5y | Night blindness | +1.25 DS/-0.5 DC×150  +1.50 DS/-0.25 DC×180 | 20/100  20/100 | AV, WDD | Severely diminished |
| 50 | *RPE65* | M | 4m-nystagmus, poor pursuit | 5y | Night blindness, SPR | -0.25 DS+4.00 DC×90  +0.25 DS+3.50 DC×90 | 20/100  20/100 | BMF | Severely diminished |
| 51 | *RDH12* | F | 6m-nystagmus | 5y | Night blindness, SPR | +2.00 DS+2.00 DC×90  +1.25 DS+1.50 DC×90 | 20/250  20/250 | Bone-spicule pigmentation, macular degeneration | Severely diminished |
| 52 | *TULP1* | M | 3m-nystagmus | 6y | Exotropia | NA | NA | NA | NA |

OD, oculus dexter; OS, oculus sinister; ERG, electroretinogram; F, female; M, male; BCVA, best corrected visual acuity; M, month(s); Y, year(s); ODS, oculo-digital sign; SPR, sluggish pupillary responses; LP, light perception; CF, counter finger; AV, attenuated vessels; BMF, bilateral mottled fundus; RPD, retinal pigment deposit; WDD, white-dot deposition; NA, not available.

Supplement Table 4 Mutation identified in 49 families with Leber congenital amaurosis

| Family number | Gene | Exon | Nucleotide change | Protein change | Allele  state | SIFT | Poly-  phen-2 | PROVEAN | Splicing AI | ACMG  Classification | Evidence level |
| --- | --- | --- | --- | --- | --- | --- | --- | --- | --- | --- | --- |
| F1 | *GUCY2D* | 12 | **c.2323C>T** | **p.Gln775*** | Het | - | - | - | - | Likely pathogenic | PVS1, PM2 |
|  |  | 17 | c.3098_3099insT | p.Tyr1035L fs*37 | Het | - | - | - | - | Uncertain | PVS1, PM4, PM2 |
| F2 | *GUCY2D* | 4 | c.1116_1119dupGGTG | p.Ser374Gly fs*74 | Hom | - | - | - | - | Pathogenic | PVS1, PS1, PM2 |
| F3 | *GUCY2D* | 3 | **c.1001T>C** | **p.Leu334Pro** | Hom | 0.005 | 0.961 | -4.41 | - | Uncertain | PM1, PM2 |
| F4 | *GUCY2D* | 2 | c.314G>A | p.Cys105Tyr | Het | 0.001 | 1 | -9.55 | - | Likely pathogenic | PM1, PM2, PM3 |
|  |  | 17 | **c.3138+2T>C** | **splicing** | Het | - | - | - | 0.72 | Uncertain | PVS1, PM4, PM2 |
| F5 | *GUCY2D* | 2 | **c.515T>C** | **p.Leu172Pro** | Het | 0.001 | 1 | -5.16 | - | Uncertain | PM2, PM5, PP3 |
|  |  | 11 | **c.2238T>A** | **p.Tyr746*** | Het | - | - | - | - | Likely pathogenic | PVS1, PM2 |
| F6 | *GUCY2D* | 3 | c.995G>C | p.Arg332Pro | Het | 0.207 | 0.463 | -1.66 | - | Uncertain | PM2 |
|  |  | 2 | c.387C>A | p.Asn129Lys | Het | 0.009 | 0.998 | -5.32 | - | Uncertain | PM2, PM3 |
| F7 | *GUCY2D* | 2 | **c.144dupC** | **P.Ala49Arg fs*270** | Het | - | - | -- | - | Likely pathogenic | PVS1, PM2 |
|  |  | 9 | c.1877C>T | p.Ser626Phe | Het | 0.000 | 0.999 | -5.87 | - | Uncertain | PM2 |
| F8 | *GUCY2D* | 12 | **c.2392_2394delATG** | **p.798_798delMet** | Het | - | - | - | - | Uncertain | PM2, PM4 |
|  |  | 15 | c.2783G>A | p.Gly928Glu | Het | 0.001 | 0.999 | -7.04 | - | Pathogenic | PM1, PM2, PM3, PM5, PP3 |
| F9-1 | *AIPL1* | 4 | **c.581_584delACGA** | **p.Tyr194Trp fs*14** | Het | - | - | - | - | Likely pathogenic | PVS1, PM2 |
|  |  | 3 | c.421C>T | p.Gln141* | Het | - | - | - | - | Pathogenic | PVS1, PM2, PM3, BS2 |
| F9-2 | *AIPL1* | 4 | **c.581_584delACGA** | **p.Tyr194Trp fs*14** | Het | - | - | - | - | Likely pathogenic | PVS1, PM2 |
|  |  | 3 | c.421C>T | p.Gln141* | Het | - | - | - | - | Pathogenic | PVS1, PM2, PM3, BS2 |
| F10 | *AIPL1* | 2 | c.247G>A | p.Glu83Lys | Het | 0.685 | 0.944 | -0.96 | - | Likely pathogenic | PM2, PM3, PP3 |
|  |  | 3 | c.421C>T | p.Gln141* | Het | - | - | - | - | Pathogenic | PVS1, PM2, PM3, BS2 |
| F11 | *AIPL1* | 3 | c.421C>T | p.Gln141* | Hom | - | - | - | - | Pathogenic | PVS1, PM2, PM3, BS2 |
| F12 | *RPGRIP1* | 4 | c.535del G | p.Glu179Ser fs*11 | Het | - | - | - | - | Pathogenic | PVS1, PM2, PM3 |
|  |  | 21 | **c.3340-1G>A** | **splicing** | Het | - | - | - | 0.98 | Likely pathogenic | PVS1, PM2 |
| F13 | *RPGRIP1* | 4 | c.535del G | p.Glu179Ser  fs*11 | Het | - | - | - | - | Pathogenic | PVS1, PM2, PM3 |
|  |  | 17 | c.2857delA | p.I953fs | Het | - | - | - | - | Likely pathogenic | PVS1, PM2 |
| F14 | *RPGRIP1* | 4 | c.535del G | p.Glu179Ser fs*11 | Hom | - | - | - | - | Pathogenic | PVS1, PM2, PM3 |
| F15 | *RPGRIP1* | 10 | **c.1305_1306delTACC** | **p.Lys435Asn fs*4** | Het | - | - | - | - | Likely pathogenic | PVS1, PM2 |
|  |  | 16 | c.2398G>A | p.Glu800Lys | Het | 0.004 | 0.709 | -3.08 | - | Uncertain | PM2, PP3 |
| F16 | *RPGRIP1* | 12 | c.1468-2A>G | splicing | Het | - | - | - | 0.8 | Pathogenic | PVS1, PM2, PM3 |
|  |  | 14 | **c.1878dupT** | **p.Leu627Ser fs*3** | Het | - | - | - | - | Likely pathogenic | PVS1, PM2 |
| F17 | *RPGRIP1* | 14 | c.2021C>A | p.Pro674His | Het | 0.001 | 1 | -6.54 | - | Pathogenic | PM1, PM2, PM3, PM5, PP3 |
|  |  | 23 | **c.3618-1G>C** | **splicing** | Het | - | - | - | 0.99 | Uncertain | PVS1, PM4, PM2 |
| F18-01 | *RPGRIP1* | 16 | **c.2422delT** | **p.Cys808Ala**  **fs*50** | Het | - | - | - | - | Likely pathogenic | PVS1, PM2 |
|  |  | 19 | c.3238+3A>G | splicing | Het | - | - | - | 0.88 | Uncertain | PM2 |
| F18-02 | *RPGRIP1* | 16 | **c.2422delT** | **p.Cys808Ala**  **fs*50** | Het | - | - | - | - | Likely pathogenic | PVS1, PM2 |
|  |  | 19 | c.3238+3A>G | splicing | Het | - | - | - | 0.88 | Uncertain | PM2 |
| F19 | *RPGRIP1* | 16 | c.2668C>T | p.Arg890* | Het | - | - | - | - | Pathogenic | PVS1, PM2, PM3 |
|  |  | 22 | c.3565C>T | p.Arg1189* | Het | - | - | - | - | Likely pathogenic | PVS1, PM4, PM2, PM3 |
| F20 | *RPGRIP1* | 17 | c.2857delA | p.Ile953Ser  fs*48 | Het | - | - | - | - | Likely pathogenic | PVS1, PM2 |
|  |  | 22 | c.3565_3571delCGAAGGC | p.Arg1189Gly  fs*7 | Het | - | - | - | - | Likely pathogenic | PVS1, PM2, PM3, PM4 |
| F21 | *RPGRIP1* |  | 1_22del | - | Het | - | - | - | - |  |  |
|  |  | 4 | c.535delG | p.Glu179Ser fs*11 | Het | - | - | - | - | Pathogenic | PVS1, PM2, PM3 |
| F22 | *RPGRIP1* | 16 | c.2592T>G | p.Tyr864* | Het | - | - | - | - | Pathogenic | PVS1, PM2, PM3 |
|  |  | 19 | c.3238+3A>G | splicing | Het | - | - | - | 0.88 | Uncertain | PM2 |
| F23 | *RPGRIP1* | 4 | c.535delG | p.Glu179Ser  fs*11 | Het | - | - | - | - | Pathogenic | PVS1, PM2, PM3 |
|  |  | 4 | c.521delC | p.Pro174Leu  fs*16 | Het | - | - | - | - | Pathogenic | PVS1, PM2, PM3 |
| F24 | *RPGRIP1* | 9 | c.964_967dupCTCC | p.Leu323Pro  fs*7 | Het | - | - | - | - | Pathogenic | PVS1, PM2, PM3 |
|  |  | 18 | c.2857delA | p.Ile953Ser  fs*48 | Het | - | - | - | - | Pathogenic | PVS1, PM2, PM3 |
| F25 | *CEP290* | 6 | c.322C>T | p.Arg108* | Het |  |  |  | - | Pathogenic | PVS1, PM2, PM3 |
|  |  | 17 | c.1666dupA | p.Ile556Asn  fs*20 | Het | - | - | - | - | Pathogenic | PVS1, PM3 |
| F26 | *CEP290* | 31 | **c.3745_3748delCTCT** | **p.Leu1249Ilefs*41** | Het | - | - | - | - | Likely pathogenic | PVS1, PM2 |
|  |  | 3 | **c.172C>G** | **p.Leu58Val** | Het | 0.189 | 0.996 | -0.3 | - | Uncertain | PM1, PM2 |
| F27 | *CEP290* | 22 | **exon22 del** | **-** | Het | - | - | - | - |  |  |
|  |  | 29 | **c.3310-1_3313delG CTTA** | **p.Leu1104Profs*14** | Het | - | - | - | - | Likely pathogenic | PVS1, PM2 |
| F28 | *CEP290* | 43 | **c.5875_5878delACTT** | **p.Thr1959Cysfs*5** | Het | - | - | - | - | Likely pathogenic | PVS1, PM2 |
|  |  | 6 | c.367C>T | p.Gln123* | Het | - | - | - | - | Pathogenic | PVS1, PM2, PM3 |
| F29 | *CEP290* | 45 | 6012-2A>G | splicing | Het | - | - | - | 0.99 | Pathogenic | PVS1, PM2, PM3 |
|  |  | 16 | 1834C>A | p.Leu612Ile | Het | - | - | - | - | Uncertain | PM2 |
| F30 | *CEP290* | 44 | c.6012G>C | p.Arg2004Ser | Het | 0.005 | 0.173 | -1.28 | - | Uncertain | PM2, PM3 |
|  |  | 44 | c.6012-2A>G | splicing | Het | - | - | - | 0.99 | Pathogenic | PVS1, PM2, PM3 |
| F31 | *CEP290* | 35 | c.4438-1G>A | splicing | Het | - | - | - | 0.99 | Pathogenic | PVS1, PM2, PM3 |
|  |  | 31 | c.3814C>T | p.Arg1272* | Het | - | - | - | - | Pathogenic | PVS1, PM2, PM3 |
| F32 | *CEP290* | 9 | **c.838_842delCATTA** | **p.His280Ser**  **fs*16** | Het | - | - | - | - | Likely pathogenic | PVS1, PM2 |
|  |  | 5 | c.367C>T | P.Gln123* | Het | - | - | - | - | Pathogenic | PVS1, PM2, PM3 |
| F33 | *CEP290* | 20 | c.1979A>T | p.Glu660Val | Het | 0.183 | 0.007 | -1.95 | - | Likely pathogenic | PM1, PM2, PM3, BP4 |
|  |  | 13 | c.1189+2T>C | splicing | Het | - | - | - | 0.99 | Likely pathogenic | PVS1, PM2 |
| F34 | *CEP290* | 13 | c.1078C>T | p.Arg360* | Het | - | - | - | - | Pathogenic | PVS1, PM2, PM3 |
|  |  | 35 | c.4661_4663delAAG | p.Glu1554del | Het | - | - | - | - | Likely pathogenic | PM2, PM3, PM4 |
| F35 | *CRB1* | 9 | c.3218T>C | p.Phe1073Ser | Hom | 0.189 | 0.999 | -0.3 | - | Likely pathogenic | PM1, PM2, PM3 |
| F36 | *CRB1* | 6 | **c.2093G>T** | **p.Cys698Phe** | Het | 0.005 | 0.999 | -1.28 | - | Likely pathogenic | PM2, PM5, PP3 |
|  |  | 11 | c.3996C>A | p.Cys1332* | Het | - | - | - | - | Likely pathogenic | PVS1, PM4, PM2, PM3 |
| F37 | *CRB1* | 7 | c.2290C>T | p.Arg764Cys | Het | 0.063 | 0.007 | -3.91 | - | Likely pathogenic | PM1, PM2, PM3 |
|  |  | 9 | **c.3112A>G** | **p.Thr1038Pro** | Het | 0.048 | 0.949 | -3.05 | - | Uncertain | PM2, BP4 |
| F38 | *CRB1* | 9 | c.3307G>A | p.Gly1103Arg | Het | 0.002 | 0.998 | -6.22 | - | Pathogenic | PS1, PM1, PM2, PM3, PM5 |
|  |  | 7 | c.2291G>A | p.Arg764His | Het | 0.128 | 0.975 | -2.32 | - | Likely pathogenic | PM1, PM2, PM3 |
| F39 | *CRX* | 4 | c.571delT | p.Tyr191Met  fs*3 | Het | - | - | - | - | Pathogenic | PVS1, PS4, PM2 |
| F40 | *LCA5* | 9 | c.1730dupT | p.Leu577fs | Het | - | - | - | - | Pathogenic | PVS1, PM2, PM3 |
|  |  | 9 | **c.1455delA** | **p.Lys485Asn**  **fs*108** | Het | - | - | - | - | Likely pathogenic | PVS1, PM2 |
| F41 | *LCA5* | 4 | **c.427C>T** | **p.Gln143*** | Het | - | - | - | - | Likely pathogenic | PVS1, PM2 |
|  |  | 9 | **c.1457_1460delACCCinsTTTTTGCCATTGTTTTGCCAT** | **p.Tyr486Phe**  **fs*113** | Het | - | - | - | - | Likely pathogenic | PVS1, PM2 |
| F42 | *LCA5* | 7 | **c.1062C>G** | **p.Tyr354*** | Hom | - | - | - | - | Pathogenic | PVS1, PM3, PM2 |
| F43-1 | *LCA5* | 9 | **c.1261C>T** | **p.Gln421*** | Hom | - | - | - | - | Pathogenic | PVS1, PM2, PM3, PP4 |
| F43-2 | *LCA5* | 9 | **c.1261C>T** | **p.Gln421*** | Hom | - | - | - | - | Pathogenic | PVS1, PM2, PM3, PP4 |
| F44 | *RPE65* | 3 | c.200T>G | p.Lys67Arg | Het | 0 | 0.951 | -4.64 | - | Pathogenic | PM1, PM2, PM3, PP3 |
|  |  | 6 | **c.596dupA** | **p.Asn199LLysfs*35** | Het | - | - | - | - | Likely pathogenic | PVS1, PM2 |
| F45 | *RPE65* | 4 | c.272G>A | p.Arg91Gln | Hom | 0.44 | 0.049 | -0.4 | - | Pathogenic | PM1, PM2, PM3, PM5, PP3 |
| F46 | *RPE65* | 6 | **c.496-1G>A** | **splicing** | Het | - | - | - | 0.97 | Likely pathogenic | PVS1, PM2 |
|  |  | 3 | c.200T>G | p.Leu67Arg | Het | 0 | 0.951 | -4.64 | - | Pathogenic | PM1, PM2, PM3, PP3 |
| F47 | *RPE65* | 6 | c.507C>A | p.Cys169* | Hom | - | - | - | - | Pathogenic | PVS1+PM2+PM3 |
| F48 | *RDH12* | 7 | c.506G>A | p.Arg169Gln | Hom | 0.001 | 1 | -4 | - | Pathogenic | PM1, PM2, PM3, PM5, PP3 |
| F49 | *TULP1* | 7 | c.627delC | p.Ser210Gln  fs*27 | Het |  |  |  |  | Pathogenic | PVS1,PM2,PM3 |
|  |  | 14 | **c.1474C>T** | **p.Gln492*** | Het |  |  |  |  | Uncertain | PVS1,PM4,PM2 |

Bold indicates novel variants. Het, heterozygote; Hom, homozygote.

Supplement Table 5 The information of the patients who conducted hemodynamic examinations.

| Patient number | Age | gene |
| --- | --- | --- |
| 04 | 6 | GUCY2D |
| 08 | 14 | GUCY2D |
| 13 | 14 | RPGRIP1 |
| 17 | 10 | RPGRIP1 |
| 24 | 6 | RPGRIP1 |
| 31 | 3 | CEP290 |
| 32 | 7 | CEP290 |
| 33 | 3 | CEP290 |
| 37 | 6 | CRB1 |
| 42 | 8 | LCA5 |
| 46 | 10 | LCA5 |
| 50 | 5 | RPE65 |

Supplement Table 6 Hemodynamic parameters in the common carotid artery, internal carotid artery, and external carotid artery in patients and compare the results with age-matched normal control subjects.

|  | common carotid artery | | | | internal carotid artery | | | | external carotid artery | | | |
| --- | --- | --- | --- | --- | --- | --- | --- | --- | --- | --- | --- | --- |
|  | PSV(cm/s) | EDV(cm/s) | PI | RI | PSV(cm/s) | EDV(cm/s) | PI | RI | PSV(cm/s) | EDV(cm/s) | PI | RI |
| LCA | 115.80±28.91 | 30.98±10.30 | 1.84±0.45 | 0.73±0.05 | 112.20±35.08 | 35.71±7.01 | 1.45±0.74 | 0.66±0.11 | 79.43±22.94 | 14.38±9.80 | 2.47±0.88 | 0.85±0.1 |
| Control | 98.31±15.38 | 25.80±2.47 | 1.77±0.25 | 0.75±0.03 | 92.10±13.40 | 34.92±3.31 | 1.16±0.23 | 0.63±0.06 | 61.30±17.35 | 10.28±5.70 | 1.75±0.57 | 0.79±0.08 |
| P | 0.13 | 0.24 | 0.67 | 0.31 | 0.23 | 0.81 | 0.36 | 0.56 | 0.12 | 0.39 | 0.11 | 0.25 |
